# The Effect of Date Palm Genotypes on Rhizobacterial Community Structures under Saline Environments

**DOI:** 10.3390/biology11111666

**Published:** 2022-11-15

**Authors:** Aya Al-Busaidi, Bernard R. Glick, Mahmoud W. Yaish

**Affiliations:** 1 Department of Biology, College of Sciences, Sultan Qaboos University, P.O. Box 36, Muscat 123, Oman; 2 Department of Biology, University of Waterloo, Waterloo, ON N2L 3G1, Canada

**Keywords:** salinity, PGPR, abiotic stress, date palm, bacterial community, Umsila, Zabad, cultivars

## Abstract

**Simple Summary:**

Soil salinity is a major global problem and negatively contributes to food production. Some date palm cultivars are tolerant to salinity, but the mechanism behind this tolerance is not yet completely understood. Microbial communities could contribute to salt tolerance traits in plants. However, the degree of contribution toward this trait is unclear in date palms. Therefore, this study aims to verify the influence of microbial communities on salinity tolerance in date palms using culture-free and next-generation DNA sequencing techniques. Upon exposure to saline conditions, changes in the microbial communities of the roots of salt-tolerant (Umsila) and susceptible (Zabad) cultivars were investigated. The results showed that the microbial communities of the cultivars are significantly different when they grow under normal soil conditions. However, when the plants grow under salinity, the microbial communities are similar. This indicates that salinity is a major factor determining the microbial community structure in date palm roots regardless of the plants’ salinity tolerance. Therefore, the date palm’s salinity tolerance is unlikely to be controlled by a special microbial community structure. This information leads scientists to focus on producing new salinity-tolerant date palms using genetics and modern molecular biology techniques rather than applying biofertilizers to existing date palm cultivars.

**Abstract:**

Some genotypes of date palms (*Phoenix dactylifera* L.) are salt-tolerant; however, salinity significantly affects others. This study aimed to determine the root epiphytic bacterial contributions to the salt tolerance mechanism in the date palm and to verify if the salt-tolerant “Umsila” and the salt-susceptible “Zabad” cultivars have different bacterial communities. Therefore, the epiphytic bacterial community structures were investigated in both cultivars when grown under control and salinity conditions. The proximal soils of the roots were collected, the DNA was extracted, and a culture-independent approach using Illumina^®^ MiSeq™ sequence analysis was carried out to identify the changes in the bacterial community structures in the soil samples due to the changes in salinity and the genotypes of the plants based on 16S rRNA gene sequencing. While salt tolerance response differences were evident between the two cultivars, the 16S rRNA gene sequencing results revealed 771 operational taxonomic units (OTUs), including 62 that were differentially accumulated in response to salinity. The ordination analysis showed significant (*p* = 0.001) changes among the communities in response to salinity in both cultivars. However, the results showed that the two cultivars had distinct bacterial communities when grown under controlled conditions, whereas they had a more similar bacterial community structure when grown under salinity conditions. The plant genotype does not affect the epiphyte bacterial community structure under salinity, probably because salinity affects the plant-microbe interaction similarly in both cultivars. Also, the identified rhizospheric bacteria are not directly associated with the root’s physiological processes in response to salinity.

## 1. Introduction

The date palm (*Phoenix dactylifera* L.) belongs to the *Arecaceae* family and is considered a halophytic plant that tolerates extreme environmental conditions, such as drought, heat, and salinity. It grows mainly in arid and semi-arid regions, such as the Middle East and North Africa [1]. Increasing soil salinity is a major problem in the Middle East, where the scarcity of rainfall and the overexploitation of groundwater results in reduced water levels in the aquifers [2]. Consequently, seawater intrudes into these aquifers, and plants that depend on them for irrigation, including date palms, are damaged [3].

Plants have a variety of salt-tolerance mechanisms that may involve bacteria [4]. These plant growth-promoting bacteria (PGPB) help host plants grow in both optimum and stressful conditions through various intricate mechanisms and subtle signaling cues that are not yet fully understood [5,6]. However, PGPB could manipulate phytohormonal signaling, enhance the solubilization of minerals, and trigger numerous other mechanisms to improve plant stress tolerance coordinately [7,8]. Therefore, some plants inoculated with PGPB strains were less negatively affected by abiotic stress because the PGPB could (at least partially) restore yield [9] by enhancing plant-water relations, ion homeostasis, and photosynthetic efficiency under saline conditions [10].

Soils contain millions to billions of bacterial cells of different types, but the colonization of plants by these bacteria depends mainly on the host plant [11,12]. Therefore, as a consequence of their genetic makeup and the subsequent composition of their root exudates, the salt-tolerant vs. susceptible cultivars of these plants may attract and host different bacterial communities [11,13]. Hence, identifying the associated microbes could be essential to understanding some of the mechanisms behind salinity tolerance or susceptibility in these plants. Therefore, in this study, two date palm cultivars of contrasting salt tolerance phenotypes, “Umsila” (salt-tolerant) and “Zabad” (salt-susceptible), were used to study the differences in the epiphytic bacterial root communities under control conditions and also in response to salt treatment. The study highlighted significant differences between the identified bacterial communities and hinted at what could occur in the rhizosphere in response to salt stress in the two date palm cultivars.

## 2. Materials and Methods

### 2.1. Soil Collections, Seed Sterilization, and Plant Growth Conditions

Soil samples were collected from the rhizospheres of date palm trees grown in a yard in the Al-Khoud area, Muscat, Oman (23°38′32.1″ N 58°11′42.8″ E). The date palm seeds of the cultivars used in this report (Umsila and Zabad) were obtained from Sultan Qaboos University’s experimental agriculture station (Muscat, Oman). The seeds were soaked in distilled water overnight to remove the fruit’s remaining tissues and were then surface-sterilized as previously described [14]. Briefly, the seeds were washed first with 75% ethanol for five minutes and then with a 0.5% hypochlorite solution (liquid bleach) for another five minutes. Subsequently, the seeds were washed with sterile water containing 10% Tween-20, and finally, they were rinsed five times with sterile distilled water. To enhance the germination of the seeds, they were soaked in distilled water for 24 h and then planted in wet, sterilized vermiculite for ten days. The germinated seeds were transferred into 4 L pots containing the collected soil. Eight pots containing natural soil were used per cultivar, including four pots for each control and a NaCl treatment with at least four seedings per pot. The NaCl-treated pots were watered with 50 mM saline water during the first week, and then the NaCl concentration was gradually increased each week by 50 mM for the first month until it reached 250 mM, which was consistently used to water the treated pots for the subsequent eight weeks. At 12 weeks following the planting, the roots were carefully removed from the pots, hand-shaken to remove the large soil particles, and brushed to collect the proximal soil around the roots.

### 2.2. Soil Physiochemical Analysis

The soil’s chemical and physical properties were measured in triplicates in the Directorate General of Agricultural and Animal Research laboratories, Ministry of Agriculture and Fisheries, Muscat, Oman. The soil from each pot was collected and stored separately. The pH was measured using a pH meter, and the electrical conductivity (EC) was measured using an Em50 Data Logger (DECAGON, Em50, 2012, Pullman, WA, USA). A calcimeter was used to measure the amount of calcium carbonate (CaCO_3_), and a flame photometer (Sherwood Scientific, 410, Cambridge, UK) was used to measure the K^+^ and Na^+^. The compounds Cl^−^, Mg^2+^, and (SO_4_)^2−^ were measured using an inductively coupled plasma optical emission spectroscopy (ICP-OES) machine (Perkin Elmer, Optima 8000, Waltham, MA, USA). The soil texture was determined with the hydrometer method based on a previously described protocol [15].

### 2.3. Gas Exchange Measurements

Before harvesting the roots for bacterial identification, gas exchange measurements were taken for the intact plant leaves. The gas exchange measurements were recorded between 8:00 and 10:00 a.m. at ambient CO_2_ concentrations. The following parameters were measured: photosynthetic rate (*A*), stomatal conductance (*gs*), transpiration rate (*E*), and intercellular CO_2_ concentration (*Ci*) using a portable photosynthesis system (LCpro-SD, ADC BioScientific, Hoddesdon, UK).

### 2.4. Root Analysis

Roots were cleaned from the attached soil, and the diameters, lengths, total surface areas of different root categories, and the number of root tips were measured using WinRHIZO software (version 5.0, Regent Instruments, Inc., Quebec, QC, Canada).

### 2.5. DNA Extraction and Illumina MiSeq Sequencing of 16S rDNA

DNA was extracted from soil samples using the Invitrogen^TM^PureLink^TM^ Genomic DNA Mini Kit, following the manufacturer’s instructions. The DNA samples’ concentrations and purity were determined using a Nanodrop2000 spectrophotometer (Thermo Scientific™, USA). All DNA samples were used to identify the bacterial community based on the 16S rRNA gene sequence analysis using high-throughput sequencing performed by Illumina^®^ MiSeq™ with the v3 reagent kit (600 cycles). The DNA sequencing procedure was conducted using ZymoBiomics Corporation, Irvine, CA, USA. The sequencing procedure was performed using >10% PhiX spike-in.

The V3-V4 DNA region of the 16S rRNA gene and the corresponding primers set were used in this analysis. The final PCR products were quantified with qPCR fluorescence readings and pooled together based on equal molarity. The final pooled library was cleaned up with a Select-a-Size DNA Clean & Concentrator™ (Zymo Research, Irvine, CA, USA), then quantified with TapeStation^®^ (Agilent Technologies, Santa Clara, CA, USA) and Qubit^®^ (Thermo Fisher Scientific, Waltham, WA, USA). Each cultivar had a total of six libraries for both treatments (control and salinity), and each treatment had three biological replicates, providing a total of 12 libraries. The ZymoBIOMICS^®^ Microbial Community DNA Standard (Zymo Research, Irvine, CA, USA) was used as a positive control for each targeted library preparation. Negative controls (i.e., a blank extraction control and blank library preparation control) were included to assess the level of bioburden carried by the wet-lab process.

### 2.6. Data Analysis

Unique PCR sequences were assumed from the raw reads using the Dada2 pipeline [16]. The chimeric sequences were removed with the Dada2 pipeline. The taxonomy assignment was performed using Uclust from Qiime v.1.9.1 [17]. Taxonomies were assigned with the Zymo Research Database, a 16S database that is internally designed and curated as a reference. The data were then further analyzed for all bacterial identification by pairwise comparisons to identify which groups were significantly different from the others using the post-hoc Tukey honest significant difference (HSD) test, using SPSS software version 21.0 [18] for *p* ≤ 0.05. Taxonomy abundance ratio bars were constructed between treatments, and cultivars were carried out to identify the dominant taxa in the control and salinity treatments. In addition, heatmaps were constructed to show the bacterial abundance profiles under different treatments using PermutMatrix software, version 1.9.3, using the default settings.

To demonstrate the variation within the bacterial communities in response to the control and salinity treatments and the type of cultivar, an ordination analysis was carried out using a non-metric dimensional scaling (N-MDS) method and the Bray-Curtis similarity index implemented within the Past 3.0 software package [19]. Finally, the phylogenetic tree was constructed with a bootstrap test (1000 replicates) using the neighbour-joinng method via Mega software package 6.0 [20].

## 3. Results

### 3.1. Soil Physiochemical Properties Affected by Salinity

The soil physicochemical properties were measured at the end of the irrigation period for both the control and NaCl-treated plants (Table 1). The results indicated that there was a significant (*p* ≤ 0.05) increase in the treated groups in comparison to the control groups, where the EC values were 0.31 dS/m (SD ± 0.05) and 7.69 dS/m (SD ± 0.49) for the soil of the control and treated “Zabad” plants, respectively, and 0.28 dS/m (SD ± 0.02) and 7.53 dS/m (SD ± 0.28), for the soil of the control and treated “Umsila” plants, respectively. Additionally, the amounts of chlorine, magnesium, and sodium significantly (*p* < 0.05) increased in the soils under the saline treatment. However, there was no significant change in the phosphorus or potassium amounts as a consequence of the salt treatment. The results indicated a significant (*p* ≤ 0.05) decrease in calcium carbonate and sulfate in the soils as a consequence of the salinity treatment. Additionally, there was a substantial decrease in the pH due to the salt treatment. The soil physical properties tests showed that the clay content increased significantly (*p* < 0.05) by approximately 2-fold under saline conditions in the soils where both cultivars were grown. However, there was no significant change in other soil properties, such as the silt, coarse, and fine sand contents for either cultivar under saline stress.

### 3.2. Salinity Causes a Significant Reduction in Plant Growth

**Figure 1:**
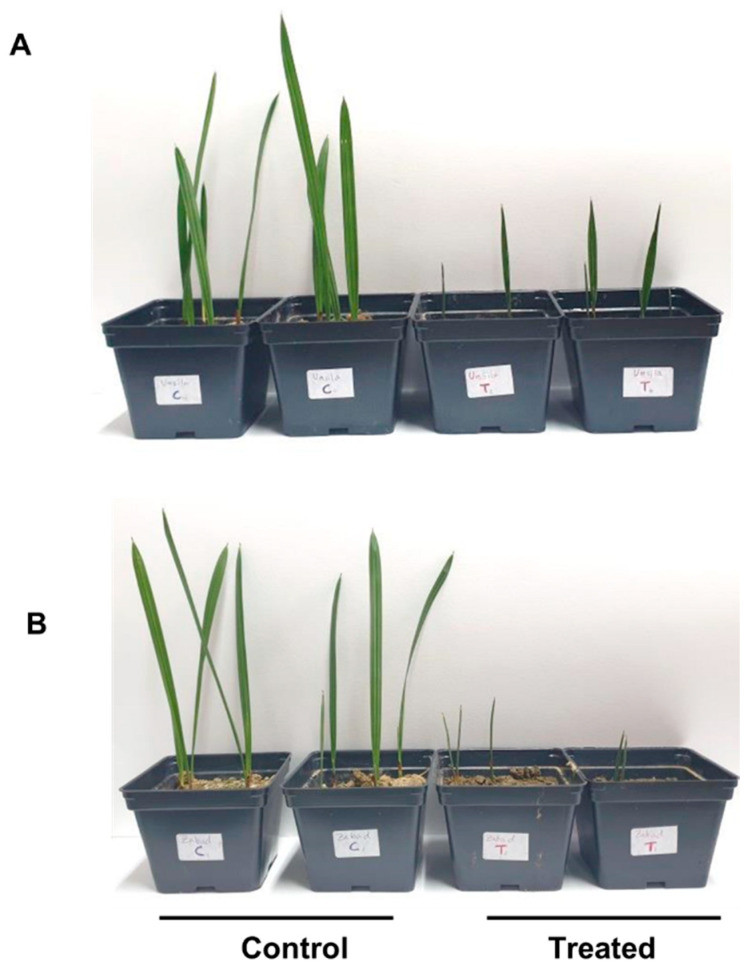
The effect of salinity on the growth of the leaves in date palm cultivars “Umsila” (**A**) and “Zabad” (**B**) when grown under control (0 mM NaCl) and treated (250 mM NaCl) conditions.

The behavior of the plants was monitored during the experiment. The results showed that the two cultivars in this experiment grew better under control conditions when compared with the NaCl-treated plants (Figure 1). The average leaf lengths were 18.10 cm (SD ± 1.75) and 17.83 cm (SD ± 2.80) for “Zabad” and “Umsila”, respectively, when they were grown under control conditions. On the other hand, the saline treatment showed a strong negative effect on both cultivars, causing stunted plants with average lengths of 5.98 cm (SD ± 1.33) and 6.42 cm (SD ± 1.98) for “Zabad” and “Umsila”, respectively.

**Figure 2:**
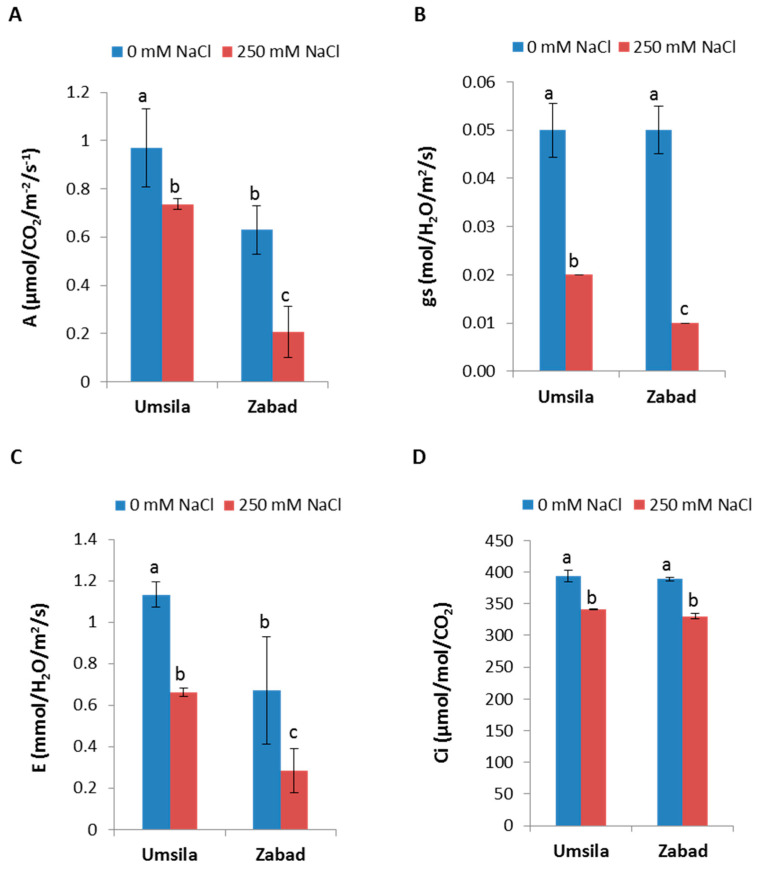
Photosynthesis parameters are affected by saline stress. The effect of salinity on the (**A**) photosynthetic rate (*A*), (**B**) stomatal conductance (*gs*), (**C**) transpiration rate (*E*), and (**D**) intercellular CO_2_ concentration (*Ci*) of cultivars “Umsila” and “Zabad” when exposed to 0 mM and 250 mM NaCl. The bars represent the mean ± SE (n = 3). Significant differences were calculated based on *p* ≤ 0.05. The bars with the same letter are not significantly different.

The health of the plants that were grown under saline soil conditions was evaluated by studying their photosynthetic performance. Generally, the results showed a consistent trend of photosynthetic deterioration due to salinity in both cultivars when the photosynthesis parameters were measured. However, the effect of salinity on “Umsila” was lower than the effect on “Zabad”. The photosynthetic rate (*A*), stomatal conductance (*gs*), transpiration rate (*E*), and intercellular CO_2_ concentration (*Ci*) all significantly (*p* ≤ 0.05) decreased in both cultivars when exposed to the salinity treatment compared to the control conditions (Figure 2). The reduction percentage in *A* was lower in “Umsila” (24%) than in “Zabad” (67%) compared with their respective controls (Figure 2A). Moreover, *gs* was lesser in “Umsila” (60%) than in “Zabad” (80%) compared with their respective controls (Figure 2B). Similarly, the percent reduction in *E* was also lesser in “Umsila” (41%) than in “Zabad” (57%) compared with their respective controls (Figure 2C). Additionally, *Ci* was lesser in “Umsila” (13%) than in “Zabad” (15%) compared with their respective controls (Figure 2D).

**Figure 3:**
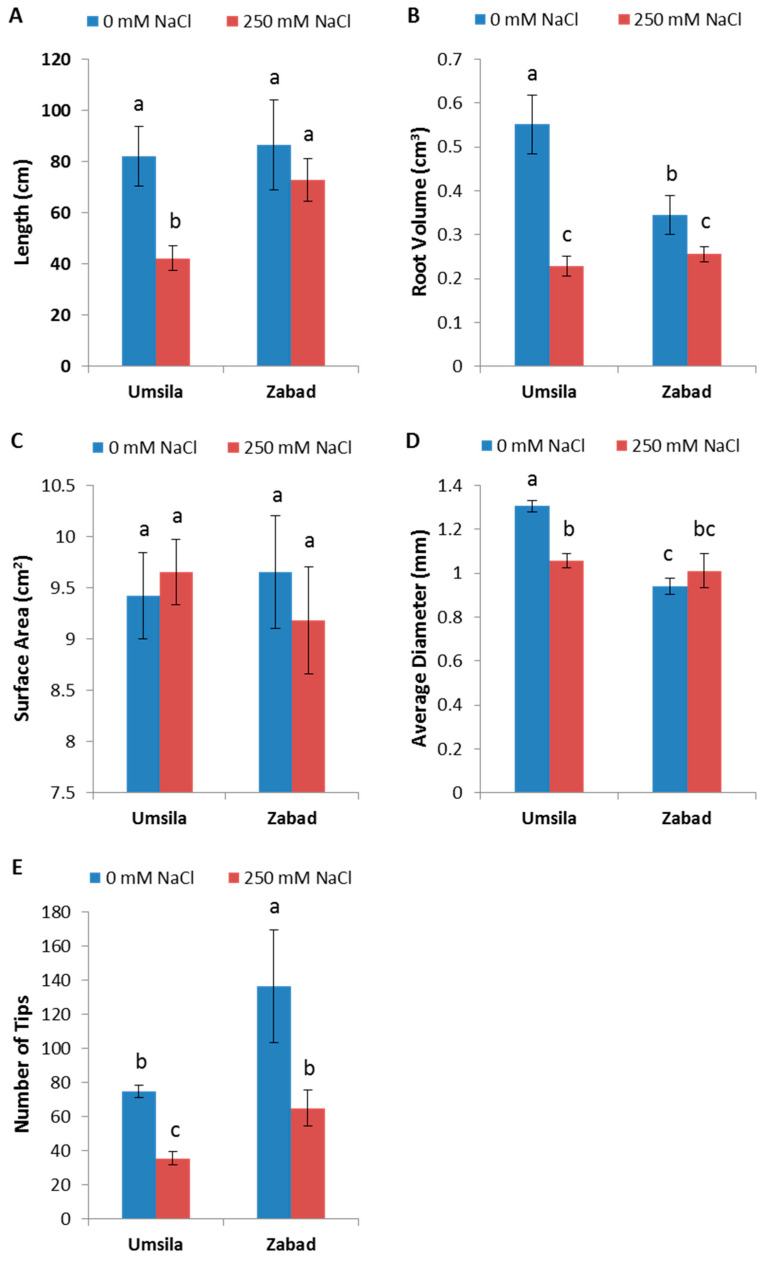
The effect of salinity on the growth of the roots. The root’s (**A**) length, (**B**) volume, (**C**) surface area, (**D**) average diameter, and (**E**) number of tips of cultivars “Umsila” and “Zabad” when exposed to 0 mM and 250 mM NaCl. The bars represent the mean ± SE (n = 4). Significant differences were calculated based on *p* ≤ 0.05. The bars with the same letter are not significantly different.

The root systems were scanned for architecture changes after the salinity treatment. The root analysis was performed by measuring the length, volume, total surface area, average diameter of roots, and the total number of root tips (Figure 3). The results showed that the root volume and the number of tips were significantly (*p* ≤ 0.05) reduced under the saline treatment for both cultivars (Figure 3B,E). In addition, unlike “Zabad”, the root lengths and the average diameter of “Umsila” were significantly (*p* ≤ 0.05) reduced under the saline treatment (Figure 3A,D). However, the surface area values did not change to any significant extent under saline stress in both cultivars (Figure 3C).

### 3.3. Metagenomic Analysis Revealed Differential Bacterial Community Structures in Different Cultivars

A total of 750,898 raw sequences were produced from the NGS sequencing of the 12 libraries of the 16S rRNA. After trimming and filtering based on the sequence size, 244,824 sequences were found to code for 5970 final unique 16S rRNA sequences. A total of 2609 high-quality reads that could be annotated and assigned as operational taxonomic units (OTUs) were obtained from both cultivars grown under control and saline conditions. Out of the 2609 reads, 76 were unidentified rRNA genes, and 1762 were not publicly available in the databases.

The results showed that the publicly unavailable reads accumulated under the saline conditions were fewer than those accumulated under the control conditions. Approximately 26% and 25.2% of these reads separately accumulated in “Umsila” and “Zabad”, respectively, under the saline conditions. However, 1.7% of them were detected in both cultivars. On the other hand, 48% and 58% of the reads were detected in “Umsila” and “Zabad”, respectively, when grown under control conditions (Supplementary Figure S1). The publicly unavailable reads showed differential accumulations. For example, the amplicons seq4, seq5, and seq18 accumulated in high amounts under salinity in both cultivars, and the amplicons seq276 and seq187 specifically accumulated under saline conditions in both cultivars.

The reads obtained from this project were filtered by removing all unidentified OTUs, leaving a total of 771 high-quality reads that were clustered into OTUs. These sequences had an average length of 416 bp.

**Figure 4:**
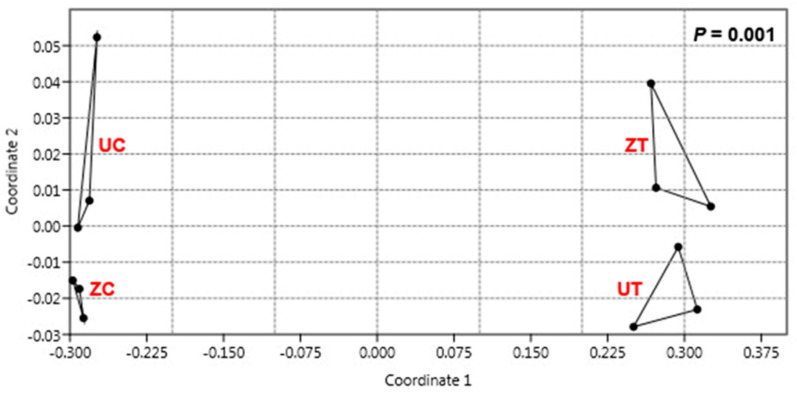
The non-metric dimensional scaling (N-MDS) analysis demonstrates distances between 771 OTUs identified from the rhizospheric bacterial communities of cultivars “Umsila” (UC) and “Zabad” (ZC) when grown under control conditions and “Umsila” (UT) and “Zabad” (ZT) cultivars when grown under salinity. The pairwise comparison using PERMANOVA showed a significant difference (*p* = 0.001) between the control and saline community groups.

Despite the low appearance of the OTUs in response to the salinity treatment, the pairwise overall variation analysis using a non-metric dimensional scaling analysis (N-MDS) and the PERMANOVA similarity test approach confirmed the appearance of a significantly (*p* = 0.001) different rhizosphere community in the date palm soil in response to salinity stress for both cultivars (Figure 4). Furthermore, the N-MDS analysis showed that the bacterial communities of the three replicates were separated based on the cultivar and the treatment type when both cultivars were under control conditions (Figure 4). However, the difference in whole bacterial community structures was remarkable under saline conditions.

The bacterial community structure of each rhizospheric cultivar was compared to identify the differentially-accumulated OTUs upon saline treatment (Supplementary Tables S1–S3). The pairwise comparison between the bacterial community structures of “Umsila” and “Zabad” under control and saline stress conditions revealed that 62 out of the 771 OTUs differentially accumulated at a significant (*p* ≤ 0.05) level in response to salinity and plant genotype. Furthermore, the annotation results revealed that 31 OTUs could be annotated and identified at the species level, while the other 31 OTUs could be determined only at the genera level.

The pairwise comparisons of the bacterial communities in “Umsila” grown under control and saline conditions revealed the presence of 20 OUTs that were differentially available in the community at significant (*p* ≤ 0.05) levels. The majority (~70%) of these OTUs (14 out of 20) were not present in “Umsila” after salinity, and 5 OTUs were uniquely found in “Umsila” after the salinity treatment (Table S1). When exposed to salinity, there were 54 differentially-accumulated OTUs in “Zabad”. Similarly, 70% of the OTUs (38) were not present following the salinity treatment. Moreover, eight unique OTUs appeared in “Zabad” when the plants were exposed to salinity (Table S2). The intra-cultivar pairwise comparison of the bacterial communities revealed that 25 OTUs were significantly (*p* ≤ 0.05) accumulated in both the “Umsila” and “Zabad” cultivars under controlled environmental conditions (Table S3). The data showed that there were 13 OTUs accumulated with varying abundances and the rest (12 OTUs) were absent in one of the cultivars under controlled conditions. Nine OTUs were not present in “Umsila”; however, only three OTUs were not in “Zabad” (Table S3). In addition, a pairwise comparison of the OTUs in both cultivars grown under saline conditions revealed the presence of only two OTUs that were differentially accumulated at significant (*p* ≤ 0.05) levels in both cultivars. The OTUs were identified as *Marinobacter guineae*, which was highly abundant in “Umsila”, and the *Pelagibacterium halotolerans*, which was highly abundant in “Zabad” due to the salinity.

### 3.4. Metagenomic Analysis Revealed Differential Bacterial Abundance in the Cultivars

**Figure 5:**
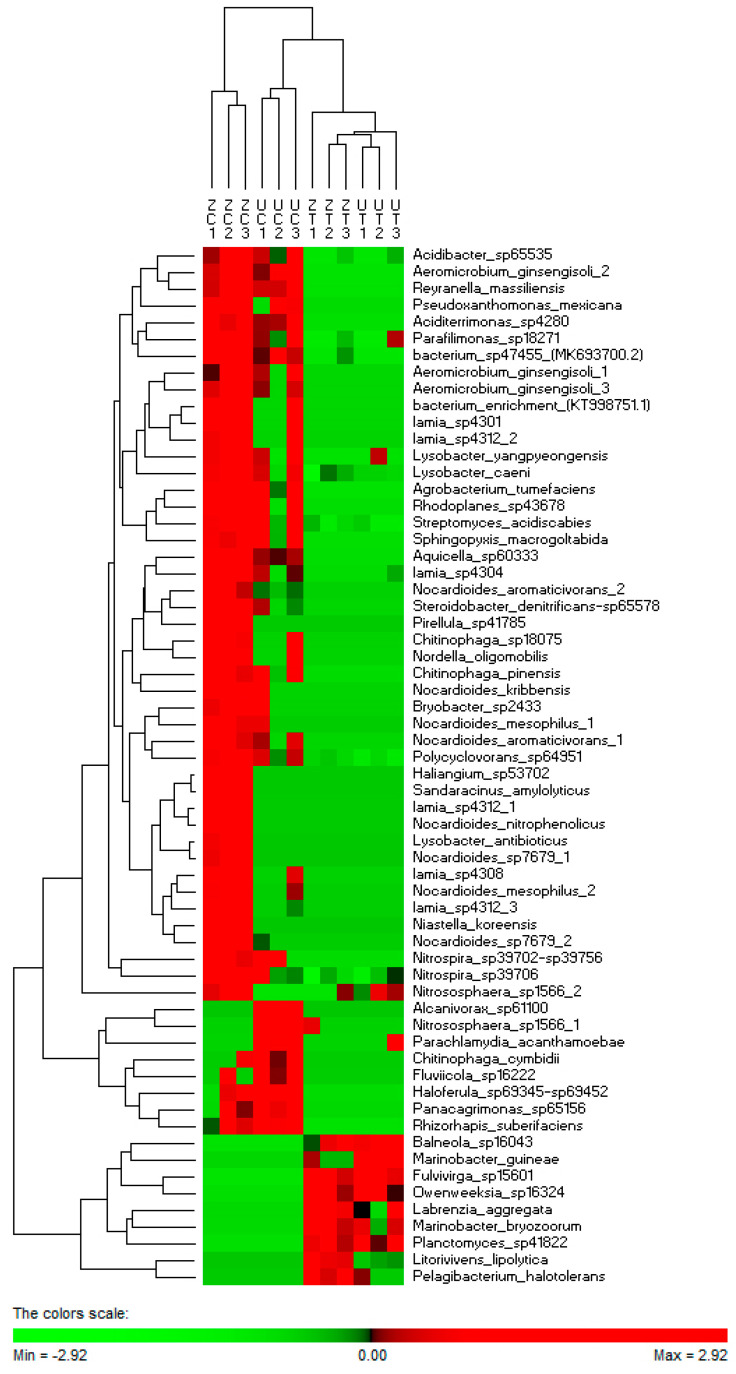
A heat map of the hierarchical cluster analysis and a dendrogram showing the normalized relative abundance of 62 OTUs identified from the rhizospheric bacterial communities of cultivars “Umsila” (UC) and “Zabad” (ZC) when grown under control conditions and cultivars “Umsila” (UT) and “Zabad” (ZT) when grown under salinity.

The hierarchical cluster analysis, based on the abundance of the OTUs identified from the “Umsila” and “Zabad” cultivars grown at the control and saline conditions, revealed that 62 OTUs were clustered into three major groups. However, *Nitrososphaera sp1566_2* did not have a consistent richness among the six replicates; therefore, it was clustered out of the first group (Figure 5). The first group included 45 OTUs with a high level of abundance in the bacterial communities identified under controlled environments. The second group had eight OTUs with a high level of abundance, mostly in “Umsila” under controlled conditions. Finally, the third group had 9 OTUs that showed bacterial communities identified mainly from saline soil with a high level of abundance.

Although these groups of OTUs shared an abundance profile among the six libraries of the same treatment (control vs. saline), the OTUs identified from “Zabad” showed a more conservative level of abundance than “Umsila” when both cultivars were grown under controlled conditions. However, both cultivars showed a more conserved abundance profile when exposed to salinity, providing evidence of the effect of salinity on the structure of the bacterial community in both cultivars.

**Figure 6:**
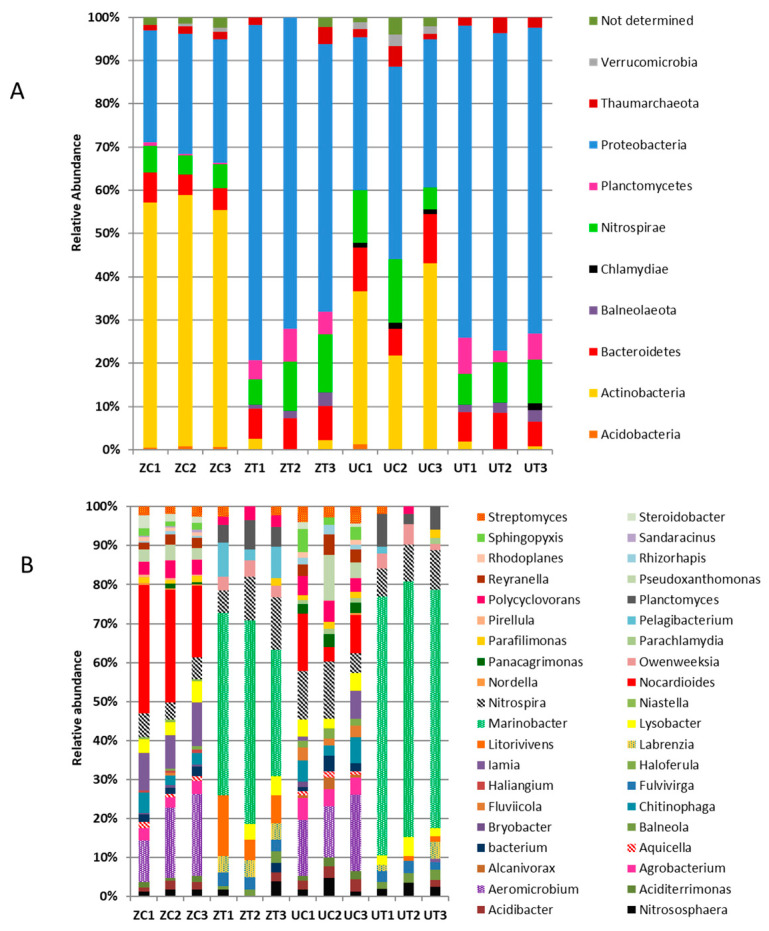
Relative abundance of the OTUs of the bacterial community (**A**) at the phylum level and (**B**) at the genus level of the cultivars “Umsila” (UC) and “Zabad” (ZC) when grown under control conditions and cultivars “Umsila” (UT) and “Zabad” (ZT) when grown under salinity. The abundance is expressed as the percentage of the total number of reads per OTU.

The relative abundance analysis of the 62 OTUs revealed the presence of 10 phyla that showed abundance under controlled and treated environments for both cultivars (Figure 6A). *Proteobacteria* was the most abundant phylum across all control and treated reads of both cultivars, forming a percentage of 52%, with 22 different genera. The *Proteobacteria* phylum was overrepresented in the salt-treated groups of both cultivars by more than double the rate compared to the control groups. It was noticed that there were trace amounts of the *Balneolaeota* phylum in the salt-treated groups, but the phylum was absent in the controls of both cultivars. Interestingly, *Planctomycetes* was overabundant in the salt-treated groups, four times more abundant than in the control groups. Moreover, *Bacteriodetes* was present across all groups at relatively low numbers (7.3%), including nine genera. The abundance of *Bacteriodetes* was slightly increased under saline stress.

On the other hand, compared to the bacteria observed during saline stress, *Actinobacteria* was overrepresented in the control groups of both of the cultivars and represented 23.1% abundance, comprising five different genera. The control conditions led to the appearance of trace amounts of *Acidobacteria*, *Chlamydiae,* and *Verrucomicrobia*. In addition, both cultivars showed relatively equal abundances for *Nitrospirae* and *Thaumarchaeota* (8.8% and 2.2%, respectively) under both control and treated conditions.

The 62 differentially-accumulated OTUs under saline conditions revealed the presence of 40 genera (Figure 6B). *Marinobacter* was the most abundant *Proteobacteria* genus under the saline stress environments for both cultivars, forming a 27% abundance which was absent under controlled environments. Only two species of *Marinobacter* were present, *M. guineae* and *M. bryozoorum.* Furthermore, eight genera were present in saline stress that were not present in controlled environments for both cultivars, including *Balneola, Fulvivirga, Labrenzia, Litorivivens, Marinobacter, Owenweeksia, Pelagibacterium,* and *Planctomyces.* On the other hand, the bacterial abundances retrieved from the rhizospheric control soil included 22 genera that were absent under saline-treated soil. Under these conditions, *Aeromicrobium* and *Nocardioides* formed the highest abundances of 8% and 9%, respectively. Other genera found under these conditions included *Aciditerrimonas*, *Agrobacterium*, *Alcanivorax*, *Aquicella*, *Bryobacter*, *Chitinophaga*, *Fluviicola*, *Haliangium*, *Haloferula*, *Nordella*, *Panacagrimonas*, *Pirellula*, *Pseudoxanthomonas*, *Reyranella*, *Rhizorhapis*, *Rhodoplanes*, *Sandaracinus*, *Sphingopyxis,* and *Steroidobacter*. *Alcanivorax* and *Parachlamydiae* were the only genera present in the rhizosphere of the “Umsila” cultivar under control conditions. *Haliangium*, *Niastella*, *Pirellula,* and *Sandaracinus* were only present in the rhizosphere of the “Zabad” cultivar under control conditions. *Nitrospira* was present across all 12 libraries at a high abundance (8.8%).

### 3.5. Salinity Affects the Structure of the Bacterial Community

**Figure 7:**
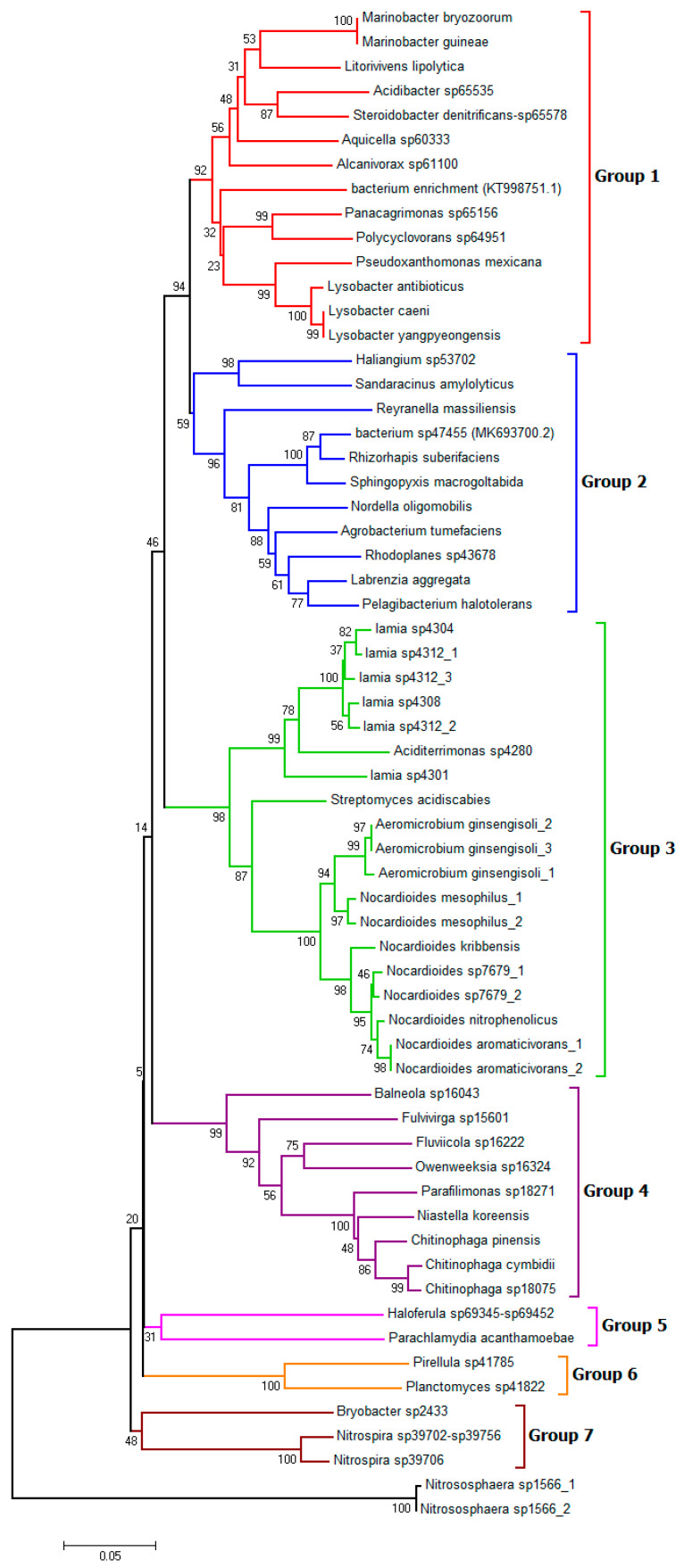
A neighbor-joining phylogenetic tree was constructed based on the 16S rRNA DNA sequences, showing the relationships between the 62 differentially-accumulated OTUs in response to salinity that were identified in this study. The bootstrap values (based on 1000 replicates) are shown at the branching node.

Based on a phylogenetic analysis, the relationship between the 16S rRNA gene sequences showed that these sequences were clustered into seven major groups (Figure 7). In groups one and two, all of the clustered OTUs belonged to the phylum *Proteobacteria*. This suggests that the bacterium sp47455 (MK693700.2) and bacterium enrichment (KT998751.1) also belonged to this phylum. All of the OTUs in group one were γ-proteobacteria. On the other hand, all group two OTUs belonged to α-proteobacteria, except for the first subtree of δ-proteobacteria. In group three, all of the OTUs were affiliated with the *Actinobacteria* phylum. Group four had clustered species of the *Bacteroidetes* phylum, except for *Balneola sp16043*, belonging to *Balneolaceae*. Group five included *Haloferula sp69345-sp69452* and *Parachlamydia acanthamoebae* that belong to *Verrucomicrobia* and *Chlamydiae*, respectively. Whereas group six included OTUs belonging only to the *Planctomycetes* phylum. Group seven contained *Acidobacteria* and *Nitrospirae* phyla. Additionally, *Nitrososphaera* sp., belonging to the *Thaumarchaeota* phylum, was not clustered within any group.

## 4. Discussion

The effect of high salinity levels on different date palm cultivars was generally severe. However, the salt-tolerant cultivar “Umsila” showed slightly less damage than the susceptible cultivar “Zabad” when the plants were grown with salinity stress in natural soil. This behavior is consistent with previous observations obtained when the cultivars were grown in artificial soil under salinity [21]. Here, the contribution of rhizospheric bacteria to salinity tolerance in the date palm was investigated. The bacterial community’s diversity and abundance depend on the soil’s nature and plant species [22]. Metagenomic analyses can provide a comprehensive understanding of the effect of salinity on the structures of rhizospheric bacterial communities in different plant genotypes. The results herein show that the plant’s bacterial community is more diverse among date palm cultivars when grown under control conditions and that this diversity is reduced when plants are exposed to salinity. Saline stress could significantly impact bacterial diversity and abundances; hence, salinity resulted in the two bacterial communities of “Zabad” and “Umsila” cultivars becoming more similar to one another than the communities found in the rhizosphere of the two cultivars when they were grown under control conditions. This observation is consistent with a study on microbial diversity in China’s desert ecosystem, where the bacterial diversity linearly decreased with increases in salinity [23]. Therefore, the N-MDS ordination analysis (Figure 4) showed that the rhizospheric communities had separated better according to saline stress than the plant’s genotype. Consistent with our results, previous studies showed that in some hypersaline soil environments, the plant genotype plays a minor role compared to soil salinity in shaping the bacterial community [24,25]. However, a host plant’s genotype was found to play a role in forming a rhizosphere community in *Arabidopsis thaliana* [26], grapevine [27], maize [28], soybean [29], and sweet potato genotypes [30].

Similar to many previous studies, the results presented in this report showed that salinity plays a crucial role in determining the OTUs identified in the rhizosphere. It was reasonably expected to find massive amounts of the *Proteobacteria* phylum across the control and treated environments for both cultivars since it is the second-largest known phylum and one of the major ones present in the soil [31]. However, the *Bacteriodetes* phylum was more abundant in the salt-treated soil. This is consistent with previously published data that showed a lesser abundance of this phylum in arable soils than in salty soils [32]. *Bacteroidetes* are copiotrophs that tend to live in environments rich in nutrients, mainly carbon, but have lower substrate affinities, a feature that might explain the slight increase in their abundance under saline stress where persistent wetting leads to disappearing aggregates in the soil and, therefore, would increase the tendency to form more soluble carbon molecules from the organic matter [33,34].

It was also noticed that most of the genera in the *Actinobacteria* phylum could not survive saline stress. Consistent with our results, a study by Xie, et al. [35] found that *Actinobacteria* tended to be abundant in salty subsurface soils but relatively reduced in saline soils (Figure 6A). Still, *Streptomyces acidiscabies*, belonging to the *Actinobacteria* phylum, a bacterium causing scab disease in potatoes, grew a nominal amount in saline conditions [36]. *Acidobacteria* disappeared in saline stress, perhaps because it has an oligotrophic property. Oligotrophs tend to live in an environment with deficient levels of nutrients and a higher substrate affinity when there is a non-limiting situation. Consistently, a study carried out by Canfora, et al. [37] showed a decreasing abundance of *Acidobacteria* as soil salinity increased. The high abundance of *Planctomycetes* in saline treatment could be explained by their aquatic nature, consistent with them being mainly found in brackish, marine, and freshwater [38,39]. The results also showed trace amounts of *Chlamydae* in the “Umsila” control libraries and in one “Zabad” treated library. According to previous studies, *Chlamydiae* tend to be more abundant in the saltwater intrusions of coastal lakes and aquifers [40].

In this study, differentially enriched OTUs identified under saline environments were previously described and isolated from saline and marine environments. For example, *Labrenzia aggregata* was identified in date palms as an endophytic differentially-accumulated bacterium under saline conditions [41]. This bacterium was isolated from marine water, which can express the *rpoN* gene that facilitates the ability to resist osmotic stress, oxidative stress, acid stress, and temperature changes [42]. However, *L. a aggregata* has not previously been reported to have growth-promoting activity in plants. The bacterium *Litorivivens lipolytica* was previously characterized from a tidal flat of the South China Sea, and *Pelagibacterium halotolerans* was identified from seawater in China [43,44]. *Marinobacter guineae* is a moderately halophilic bacterium previously isolated from Antarctica [45] and was recently reported as a potential source of long-chain polyunsaturated fatty acids [46]. A bacterial strain of the genus *Marinobacter* i.e., *Marinobacter algicola* is an endophytic bacterium previously identified in date palms under saline conditions [41]. *Marinobacter guineae* was specifically accumulated in the “Umsila” cultivar under saline conditions. Therefore, *Marinobacter* spp. may be important in the salt tolerance mechanism of this plant; nevertheless, these bacteria were not previously reported as a plant growth-promoting species.

The data showed several previously characterized OTUs that were also differentially enriched under controlled environments. For instance, *Rhizorhapis suberifaciens* was previously isolated from lettuce and was found to cause corky root disease [47]. Additionally, six characterized species of the gram-positive bacterium *Nocardioides* were also present under controlled environments. *N. aromaticivorans* can degrade the organic compounds dibenzofuran and carbazole [48]. Furthermore, *Alcanivorax sp61100* was the only differentially enriched species found in the “Umsila” cultivar under control conditions. *A. sp61100* is a halophilic, marine, oil-degrading bacterium found in low abundance in unpolluted areas and the top layers of the ocean [49]. Equally important, *Haliangium* sp. is one of the differentially enriched species in the “Zabad” cultivar under control conditions. Finally, the common rhizobacteria found in both cultivars may represent a potential “core rhizospheric microbiota” irrespective of the host plant’s inherent characteristics.

## 5. Conclusions

Date palm genotype significantly affected the proximal rhizospheric bacterial community structures under control conditions. However, salinity was more effective in controlling the bacterial community than the plant genotype. That is likely because salinity could also control the rhizospheric environment in both cultivars regardless of the differences in the plant’s physiological responses to saline stresses. The study showed a few potentially influential OTUs in salt tolerance mechanisms, such as *Marinobacter guineae*. However, the capacity of these OTUs to promote date palm growth under saline conditions must be verified empirically. The results obtained from this study provide insights into the rhizospheric bacterial community distribution among different cultivars and show that the bacterial communities might contribute to salinity tolerance in date palms. Based on these results, we believe that the rhizospheric bacterial community does not have a major role in the salt tolerance of date palms, and the plant’s genomic makeup is the primary source of knowledge required to explore and decipher salinity tolerance mechanisms in date palms.

## Figures and Tables

**Table 1:** Physiochemical properties of the soil used to plant the “Umsila” cultivar under control (UC) and saline (UT) conditions and the “Zabad” cultivar under control (ZC) and saline (ZT) conditions. The statistical analysis was conducted using a *t*-test; mean ± SD, n = 3.

Soil Physiochemical Properties	Average Content		*p*-Value	Average Content		*p*-Value
	UC	UT		ZC	ZT	
EC (dS/m)	4.37	62.77	0	4.35	59.9	0
pH	8.01	7.54	0	7.93	7.41	0
P (ppm)	156.47	100.67	0.07	127.37	118.1	0.25
K (ppm)	136.67	133.33	0.26	116.67	123.33	0.27
Na (ppm)	886.67	3620	0	620	3670	0
Coarse sand (%)	12.67	13.25	0.19	9.08	9.98	0.31
Fine sand (%)	54.37	51.78	0.13	57.95	54.39	0.16
Silt (%)	29.33	26	0.13	27.33	24.67	0.22
Clay (%)	3.63	8.97	0.02	5.63	10.97	0.03
CaCO_3_ (%)	20.93	19.9	0.04	18.73	16.97	0.04
Cl (ppm)	498.96	19.721.26	0	496.86	20.887.83	0
SO_4_ (ppm)	438.71	337.48	0.031	457.66	391.30	0.14
Mg (ppm)	338.73	1791.12	0	331.77	1510.70	0

## Data Availability

The metagenomic data produced in this report were submitted to GenBank as a sequence read archive (SRA) under the SUB12013099 code.

## Supplementary Materials

The following supporting information can be downloaded at: https://www.mdpi.com/article/10.3390/biology11111666/s1, Table S1: Differentially-accumulated OTUs in “Umsila” due to salinity treatment. The OTUs were identified from the “Umsila” cultivar when it was grown under control (UC) and salinity (UT) conditions. Table S2: Differentially-accumulated OTUs in “Zabad” due to the salinity treatment. The OTUs were identified from the “Zabad” cultivar when it was grown under control (ZC) and salinity (ZT) conditions. Table S3: Differentially-accumulated OTUs in “Umsila” and “Zabad” under control conditions. The OTUs were identified from “Umsila” (UT) and “Zabad” (ZT) when grown under salt conditions. Figure S1: A heat map of the hierarchical cluster analysis and a dendrogram showing the normalized relative abundance of publicly unavailable reads identified from the rhizospheric bacterial communities of cultivars “Umsila” (UC) and “Zabad” (ZC) when grown under control conditions and cultivars “Umsila” (UT) and “Zabad” (ZT) when grown under salinity.
