# Supplementary material for: The Effect of Date Palm Genotypes on Rhizobacterial Community Structures under Saline Environments
Source: Biology (Basel). 2022 Nov 15;11(11):1666. doi: 10.3390/biology11111666 (PMC9687558; doi:10.3390/biology11111666)
Supplement: Supplementary file 1 [file biology-11-01666-s001.zip › Supplimentary Figure 1.pdf]

Rows : - Linkage rule: McQuitty's criteria
- Tree Seriation rule: Multiple-fragment heuristic (MF)

Columns : - Linkage rule: McQuitty's criteria
- Tree Seriation rule: Multiple-fragment heuristic (MF)

Dissimilarity : - Euclidean distance

The colors scale:

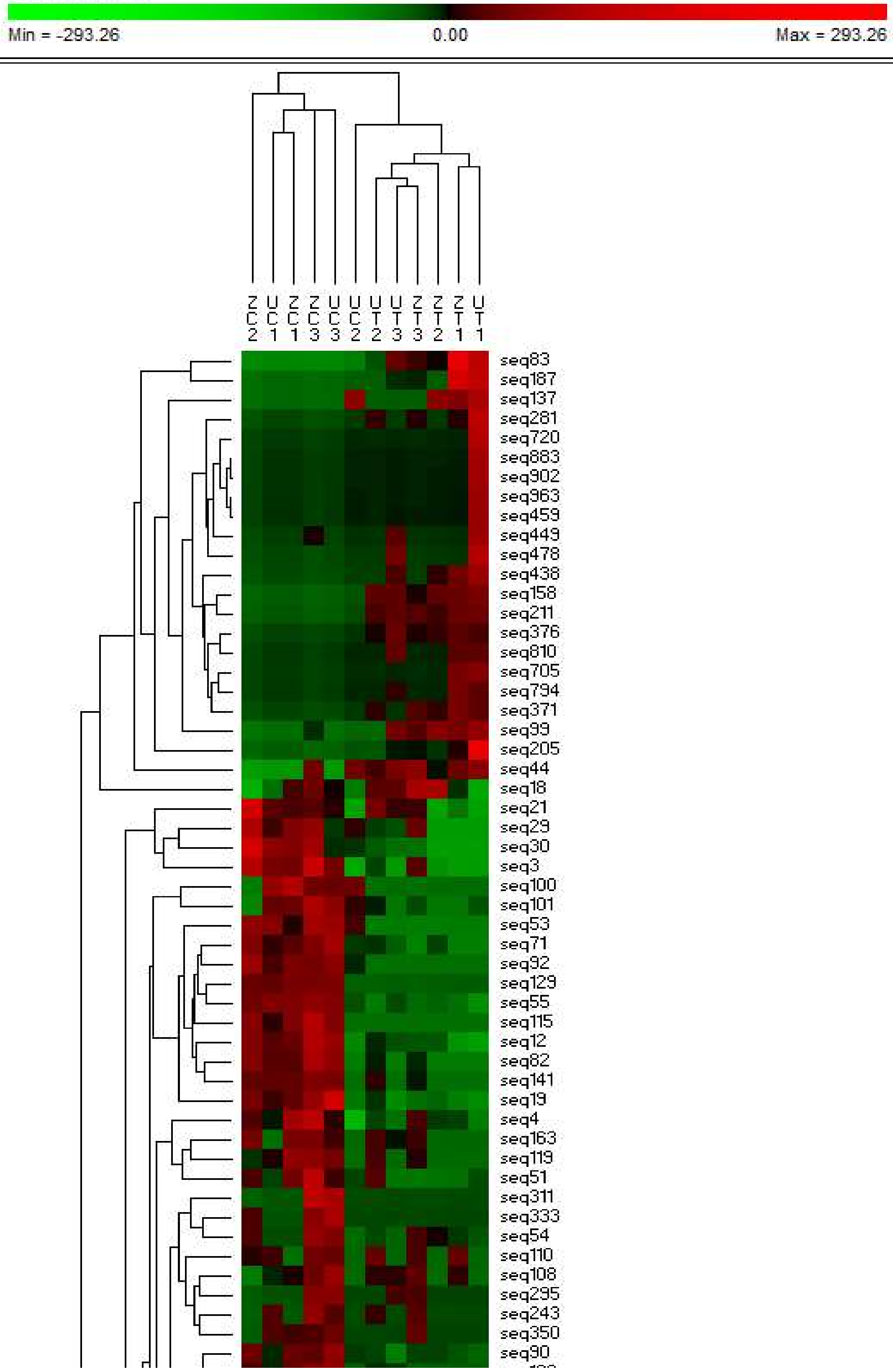

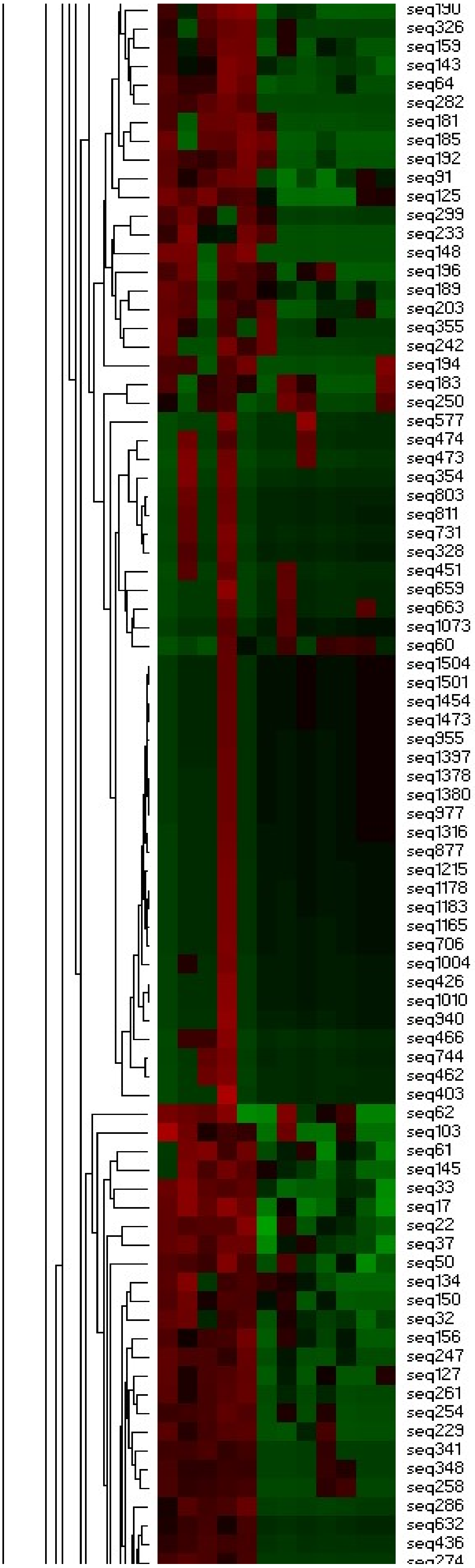
seq326
seq159
seq143
seq64
seq282
seq181
seq185
seq192
seq91
seq125
seq299
seq233
seq148
seq196
seq189
seq203
seq355
seq242
seq194
seq183
seq250
seq577
seq474
seq473
seq354
seq803
seq811
seq731
seq328
seq451
seq659
seq663
seq1073
seq60
seq1504
seq1501
seq1454
seq1473
seq955
seq1397
seq1378
seq1380
seq977
seq1316
seq877
seq1215
seq1178
seq1183
seq1165
seq706
seq1004
seq426
seq1010
seq940
seq466
seq744
seq462
seq403
seq62
seq103
seq61
seq145
seq33
seq17
seq22
seq37
seq50
seq134
seq150
seq32
seq156
seq247
seq127
seq261
seq254
seq229
seq341
seq348
seq258
seq286
seq632
seq436

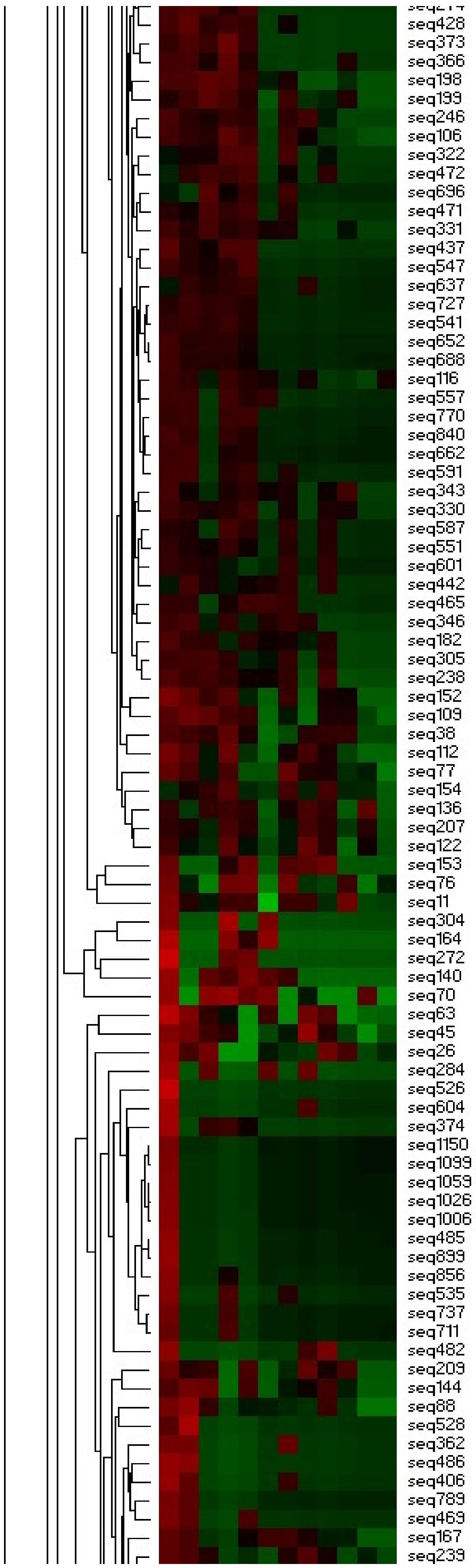
seq428
seq373
seq366
seq198
seq199
seq246
seq106
seq322
seq472
seq696
seq471
seq331
seq437
seq547
seq637
seq727
seq541
seq652
seq688
seq116
seq557
seq770
seq840
seq662
seq591
seq343
seq330
seq587
seq551
seq601
seq442
seq465
seq346
seq182
seq305
seq238
seq152
seq109
seq38
seq112
seq77
seq154
seq136
seq207
seq122
seq153
seq76
seq11
seq304
seq164
seq272
seq140
seq70
seq63
seq45
seq26
seq284
seq526
seq604
seq374
seq1150
seq1099
seq1059
seq1026
seq1006
seq485
seq899
seq856
seq535
seq737
seq711
seq482
seq209
seq144
seq88
seq528
seq362
seq486
seq406
seq789
seq469
seq167
seq239

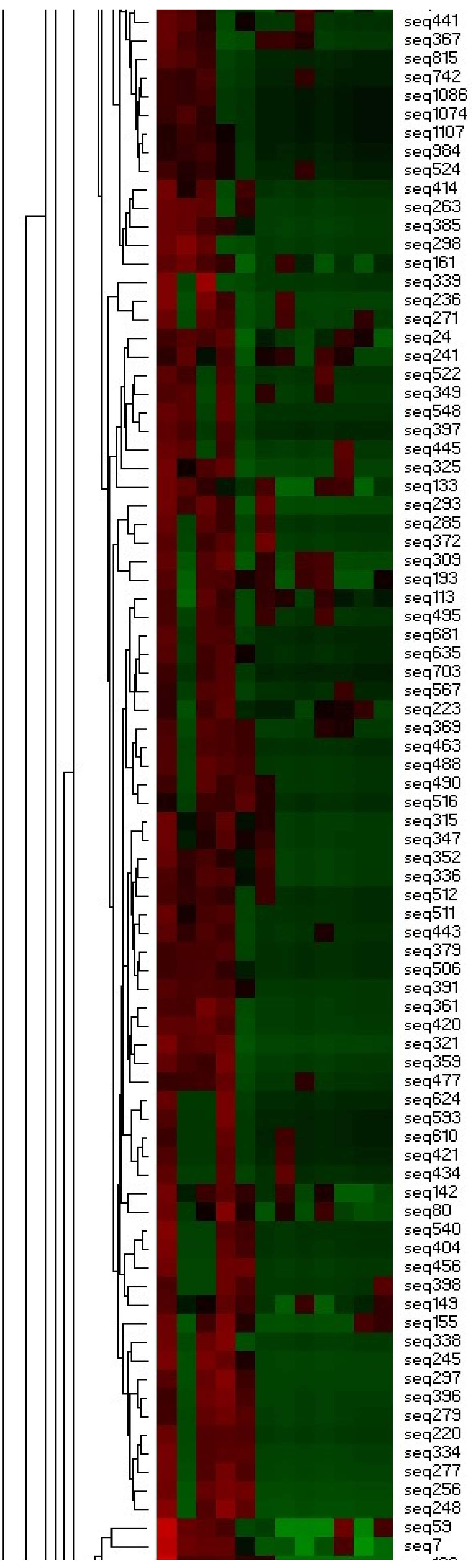
seq441
seq367
seq815
seq742
seq1086
seq1074
seq1107
seq984
seq524
seq414
seq263
seq385
seq298
seq161
seq339
seq236
seq271
seq24
seq241
seq522
seq349
seq548
seq397
seq445
seq325
seq133
seq293
seq285
seq372
seq309
seq193
seq113
seq495
seq681
seq635
seq703
seq567
seq223
seq369
seq463
seq488
seq490
seq516
seq315
seq347
seq352
seq336
seq512
seq511
seq443
seq379
seq506
seq391
seq361
seq420
seq321
seq359
seq477
seq624
seq593
seq610
seq421
seq434
seq142
seq80
seq540
seq404
seq456
seq398
seq149
seq155
seq338
seq245
seq297
seq396
seq279
seq220
seq334
seq277
seq256
seq248
seq59
seq7

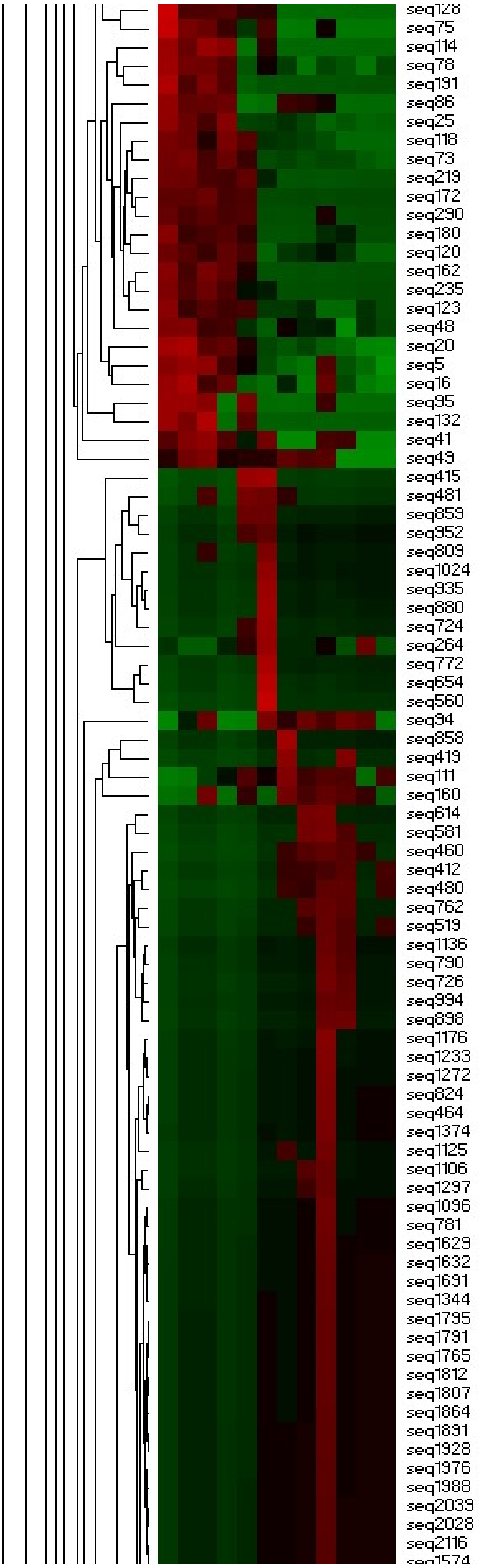
seq128
seq75
seq114
seq78
seq191
seq86
seq25
seq118
seq73
seq219
seq172
seq290
seq180
seq120
seq162
seq235
seq123
seq48
seq20
seq5
seq16
seq95
seq132
seq41
seq49
seq415
seq481
seq859
seq952
seq809
seq1024
seq935
seq880
seq724
seq264
seq772
seq654
seq560
seq94
seq858
seq419
seq111
seq160
seq614
seq581
seq460
seq412
seq480
seq762
seq519
seq1136
seq790
seq726
seq994
seq898
seq1176
seq1233
seq1272
seq824
seq464
seq1374
seq1125
seq1106
seq1297
seq1096
seq781
seq1629
seq1632
seq1691
seq1344
seq1795
seq1791
seq1765
seq1812
seq1807
seq1864
seq1891
seq1928
seq1976
seq1988
seq2039
seq2028
seq2116

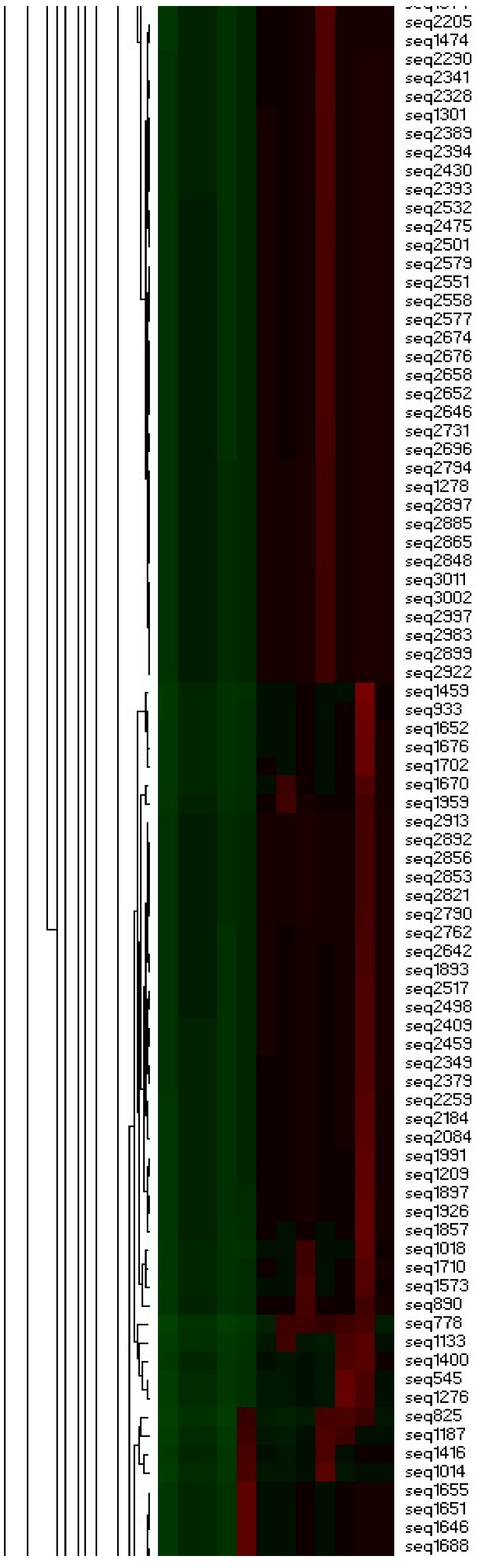
seq2205
seq1474
seq2290
seq2341
seq2328
seq1301
seq2389
seq2394
seq2430
seq2393
seq2532
seq2475
seq2501
seq2579
seq2551
seq2558
seq2577
seq2674
seq2676
seq2658
seq2652
seq2646
seq2731
seq2696
seq2794
seq1278
seq2897
seq2885
seq2865
seq2848
seq3011
seq3002
seq2997
seq2983
seq2899
seq2922
seq1459
seq933
seq1652
seq1676
seq1702
seq1670
seq1959
seq2913
seq2892
seq2856
seq2853
seq2821
seq2790
seq2762
seq2642
seq1893
seq2517
seq2498
seq2409
seq2459
seq2349
seq2379
seq2259
seq2184
seq2084
seq1991
seq1209
seq1897
seq1926
seq1857
seq1018
seq1710
seq1573
seq890
seq778
seq1133
seq1400
seq545
seq1276
seq825
seq1187
seq1416
seq1014
seq1655
seq1651
seq1646
seq1688

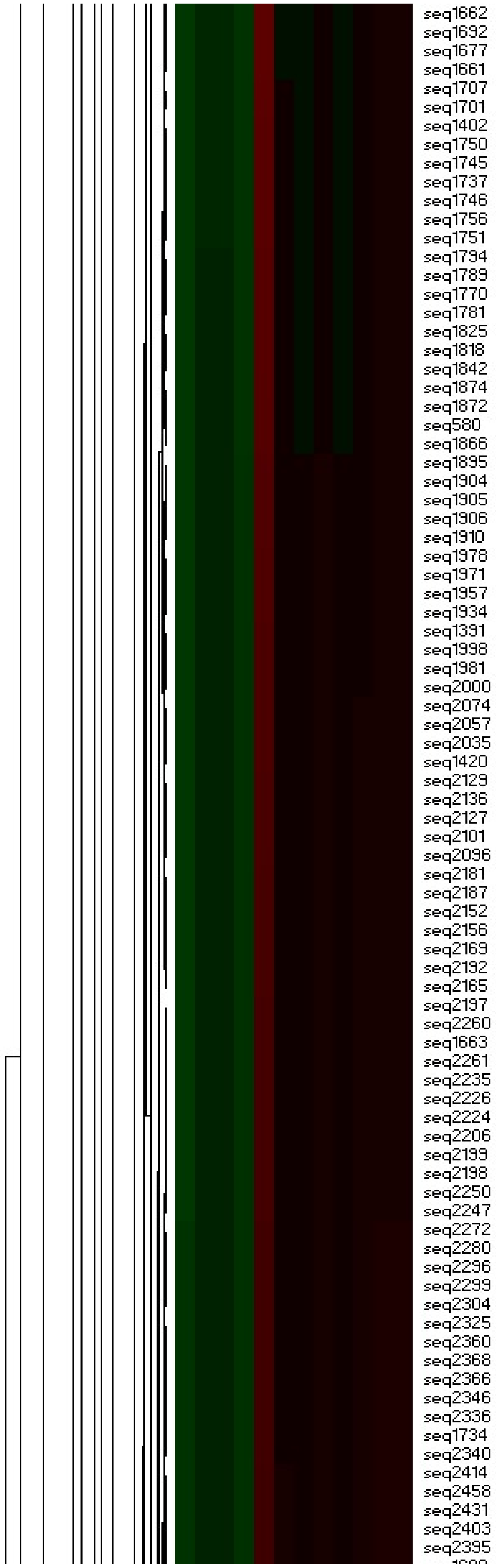
seq1662
seq1692
seq1677
seq1661
seq1707
seq1701
seq1402
seq1750
seq1745
seq1737
seq1746
seq1756
seq1751
seq1794
seq1789
seq1770
seq1781
seq1825
seq1818
seq1842
seq1874
seq1872
seq580
seq1866
seq1895
seq1904
seq1905
seq1906
seq1910
seq1978
seq1971
seq1957
seq1934
seq1391
seq1998
seq1981
seq2000
seq2074
seq2057
seq2035
seq1420
seq2129
seq2136
seq2127
seq2101
seq2096
seq2181
seq2187
seq2152
seq2156
seq2169
seq2192
seq2165
seq2197
seq2260
seq1663
seq2261
seq2235
seq2226
seq2224
seq2206
seq2199
seq2198
seq2250
seq2247
seq2272
seq2280
seq2296
seq2299
seq2304
seq2325
seq2360
seq2368
seq2366
seq2346
seq2336
seq1734
seq2340
seq2414
seq2458
seq2431
seq2403
seq2395

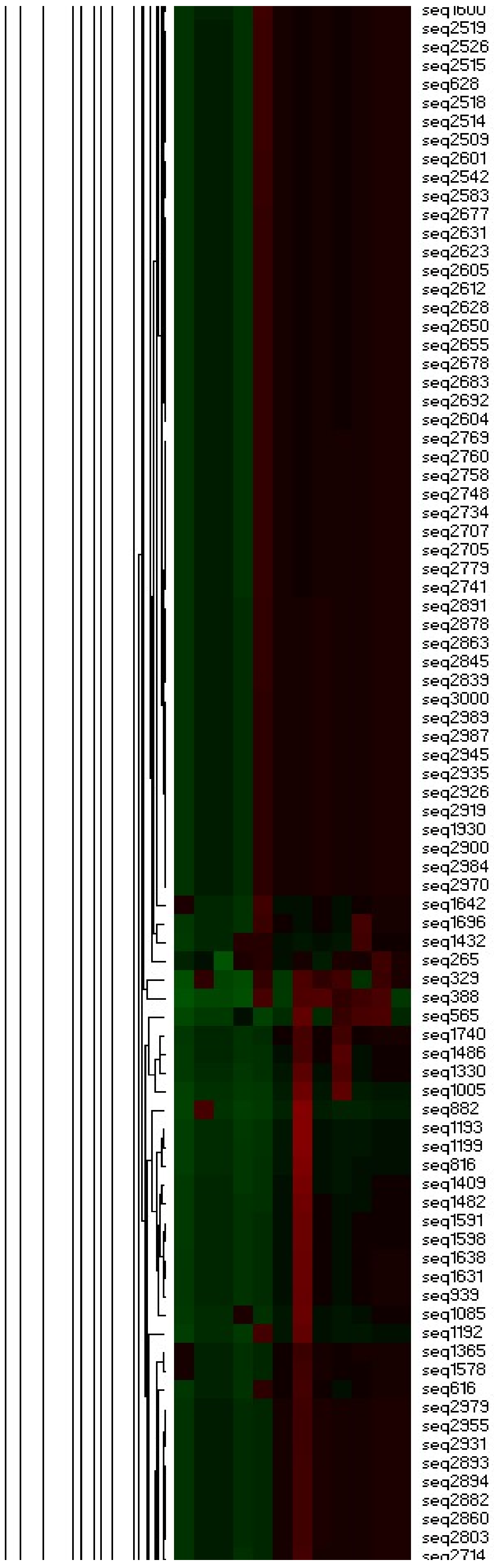
seq1600
seq2519
seq2526
seq2515
seq628
seq2518
seq2514
seq2509
seq2601
seq2542
seq2583
seq2677
seq2631
seq2623
seq2605
seq2612
seq2628
seq2650
seq2655
seq2678
seq2683
seq2692
seq2604
seq2769
seq2760
seq2758
seq2748
seq2734
seq2707
seq2705
seq2779
seq2741
seq2891
seq2878
seq2863
seq2845
seq2839
seq3000
seq2989
seq2987
seq2945
seq2935
seq2926
seq2919
seq1930
seq2900
seq2984
seq2970
seq1642
seq1696
seq1432
seq265
seq329
seq388
seq565
seq1740
seq1486
seq1330
seq1005
seq882
seq1193
seq1199
seq816
seq1409
seq1482
seq1591
seq1598
seq1638
seq1631
seq939
seq1085
seq1192
seq1365
seq1578
seq616
seq2979
seq2955
seq2931
seq2893
seq2894
seq2882
seq2860
seq2803
seq2714

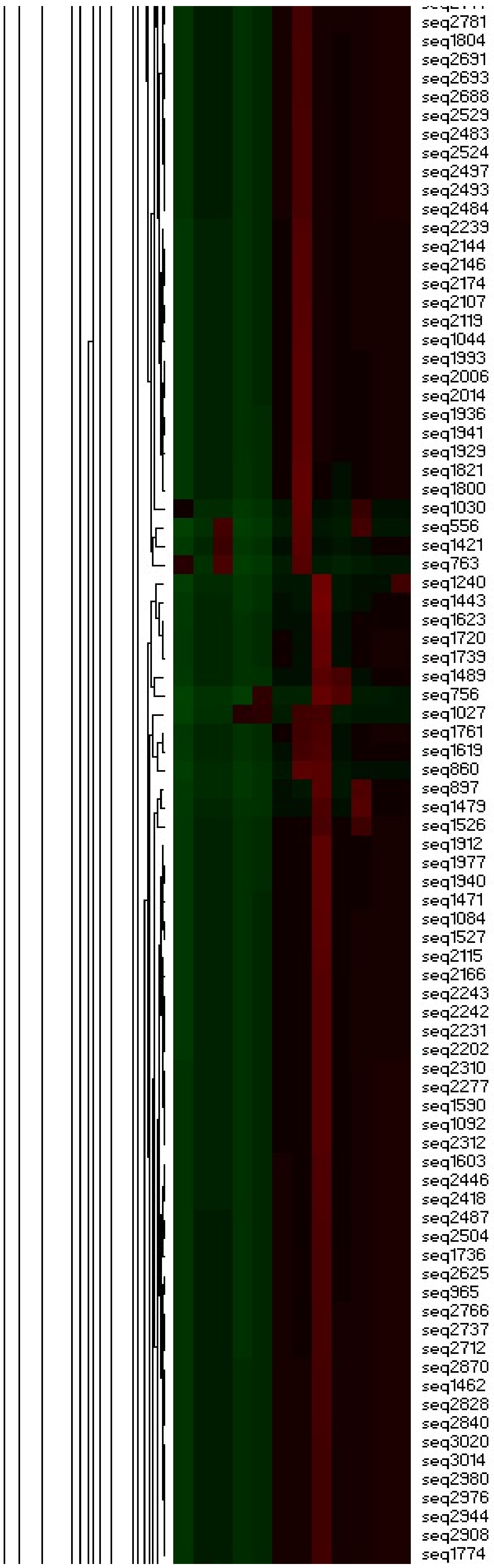
seq2781
seq1804
seq2691
seq2693
seq2688
seq2529
seq2483
seq2524
seq2497
seq2493
seq2484
seq2239
seq2144
seq2146
seq2174
seq2107
seq2119
seq1044
seq1993
seq2006
seq2014
seq1936
seq1941
seq1929
seq1821
seq1800
seq1030
seq556
seq1421
seq763
seq1240
seq1443
seq1623
seq1720
seq1739
seq1489
seq756
seq1027
seq1761
seq1619
seq860
seq897
seq1479
seq1526
seq1912
seq1977
seq1940
seq1471
seq1084
seq1527
seq2115
seq2166
seq2243
seq2242
seq2231
seq2202
seq2310
seq2277
seq1590
seq1092
seq2312
seq1603
seq2446
seq2418
seq2487
seq2504
seq1736
seq2625
seq965
seq2766
seq2737
seq2712
seq2870
seq1462
seq2828
seq2840
seq3020
seq3014
seq2980
seq2976
seq2944
seq2908
seq1774

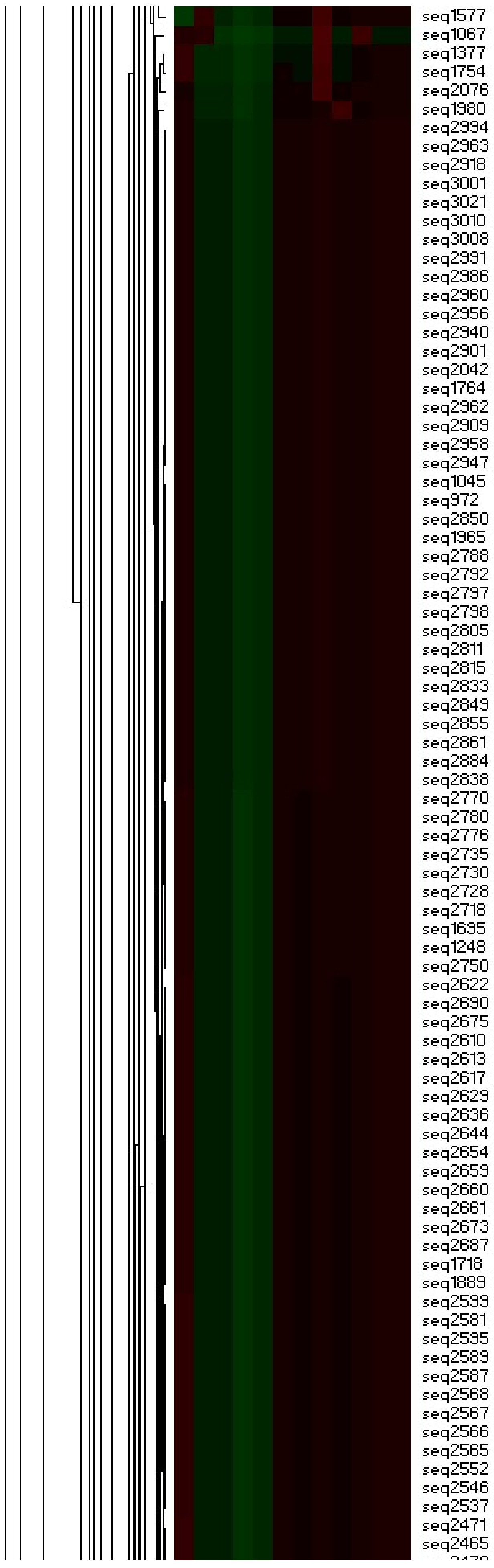
seq1577
seq1067
seq1377
seq1754
seq2076
seq1980
seq2994
seq2963
seq2918
seq3001
seq3021
seq3010
seq3008
seq2991
seq2986
seq2960
seq2956
seq2940
seq2901
seq2042
seq1764
seq2962
seq2909
seq2958
seq2947
seq1045
seq972
seq2850
seq1965
seq2788
seq2792
seq2797
seq2798
seq2805
seq2811
seq2815
seq2833
seq2849
seq2855
seq2861
seq2884
seq2838
seq2770
seq2780
seq2776
seq2735
seq2730
seq2728
seq2718
seq1695
seq1248
seq2750
seq2622
seq2690
seq2675
seq2610
seq2613
seq2617
seq2629
seq2636
seq2644
seq2654
seq2659
seq2660
seq2661
seq2673
seq2687
seq1718
seq1889
seq2599
seq2581
seq2595
seq2589
seq2587
seq2568
seq2567
seq2566
seq2565
seq2552
seq2546
seq2537
seq2471
seq2465

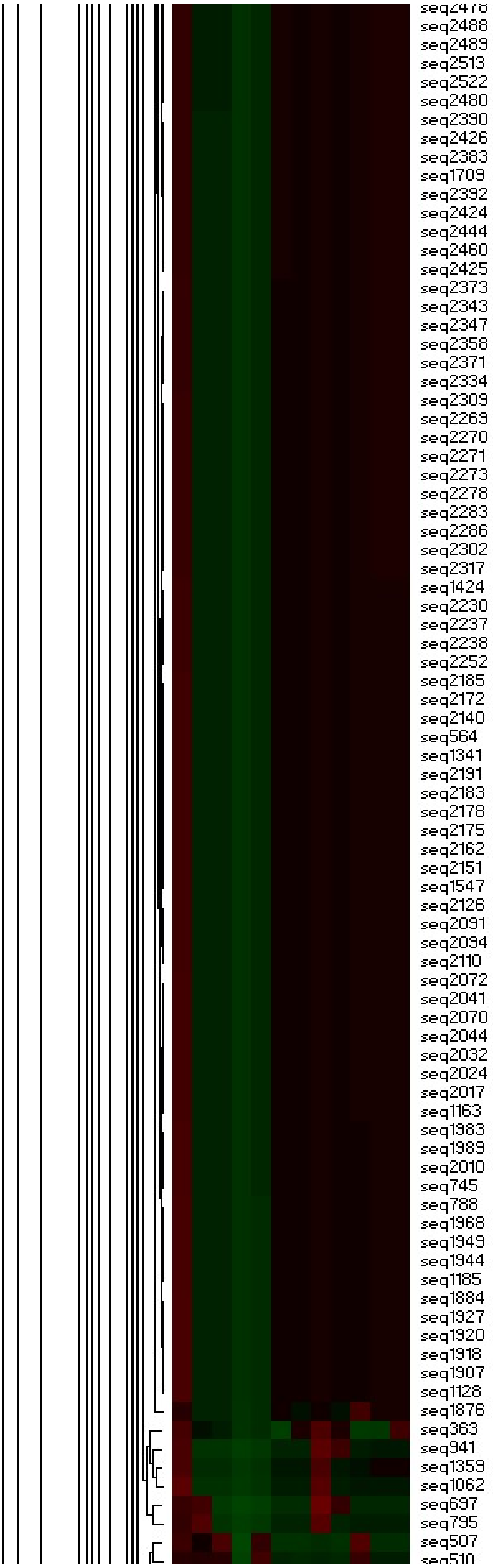
seq2478
seq2488
seq2489
seq2513
seq2522
seq2480
seq2390
seq2426
seq2383
seq1709
seq2392
seq2424
seq2444
seq2460
seq2425
seq2373
seq2343
seq2347
seq2358
seq2371
seq2334
seq2309
seq2269
seq2270
seq2271
seq2273
seq2278
seq2283
seq2286
seq2302
seq2317
seq1424
seq2230
seq2237
seq2238
seq2252
seq2185
seq2172
seq2140
seq564
seq1341
seq2191
seq2183
seq2178
seq2175
seq2162
seq2151
seq1547
seq2126
seq2091
seq2094
seq2110
seq2072
seq2041
seq2070
seq2044
seq2032
seq2024
seq2017
seq1163
seq1983
seq1989
seq2010
seq745
seq788
seq1968
seq1949
seq1944
seq1185
seq1884
seq1927
seq1920
seq1918
seq1907
seq1128
seq1876
seq363
seq941
seq1359
seq1062
seq697
seq795
seq507
seq510

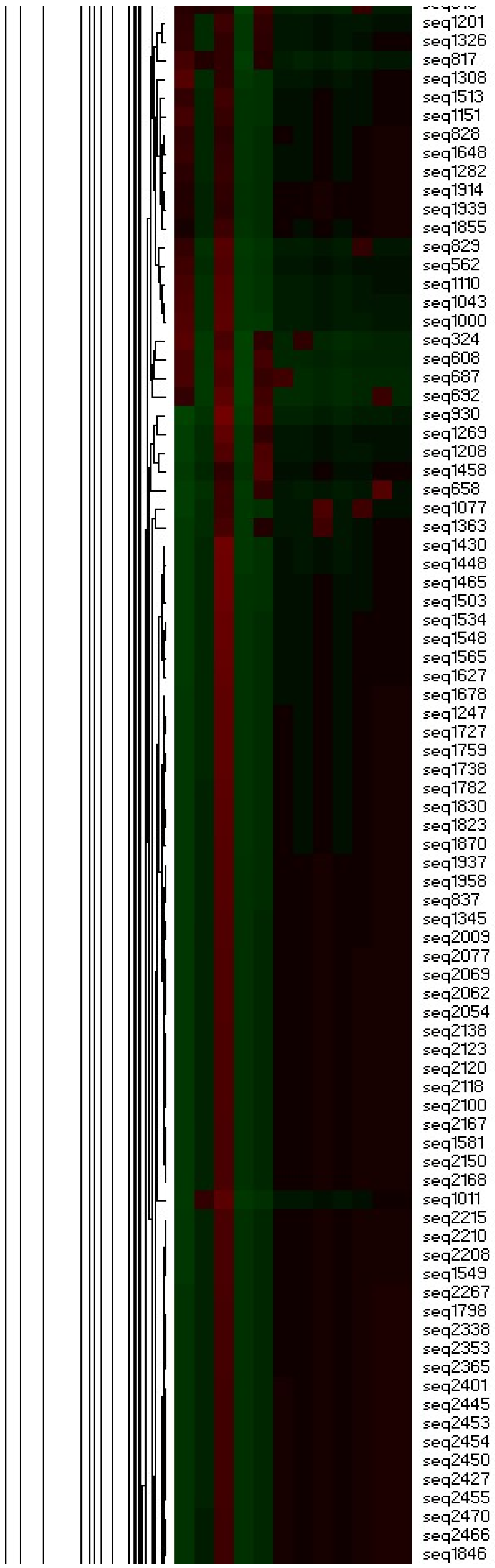
seq1201
seq1326
seq817
seq1308
seq1513
seq1151
seq828
seq1648
seq1282
seq1914
seq1939
seq1855
seq829
seq562
seq1110
seq1043
seq1000
seq324
seq608
seq687
seq692
seq930
seq1269
seq1208
seq1458
seq658
seq1077
seq1363
seq1430
seq1448
seq1465
seq1503
seq1534
seq1548
seq1565
seq1627
seq1678
seq1247
seq1727
seq1759
seq1738
seq1782
seq1830
seq1823
seq1870
seq1937
seq1958
seq837
seq1345
seq2009
seq2077
seq2069
seq2062
seq2054
seq2138
seq2123
seq2120
seq2118
seq2100
seq2167
seq1581
seq2150
seq2168
seq1011
seq2215
seq2210
seq2208
seq1549
seq2267
seq1798
seq2338
seq2353
seq2365
seq2401
seq2445
seq2453
seq2454
seq2450
seq2427
seq2455
seq2470
seq2466
seq1846

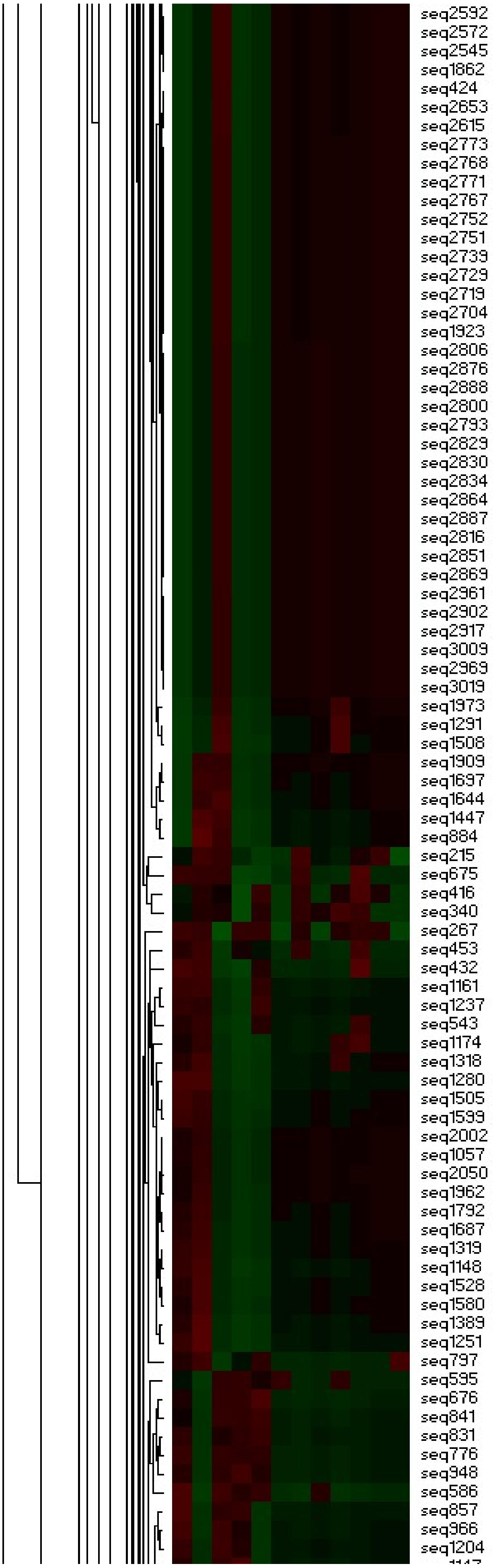
seq2592
seq2572
seq2545
seq1862
seq424
seq2653
seq2615
seq2773
seq2768
seq2771
seq2767
seq2752
seq2751
seq2739
seq2729
seq2719
seq2704
seq1923
seq2806
seq2876
seq2888
seq2800
seq2793
seq2829
seq2830
seq2834
seq2864
seq2887
seq2816
seq2851
seq2869
seq2961
seq2902
seq2917
seq3009
seq2969
seq3019
seq1973
seq1291
seq1508
seq1909
seq1697
seq1644
seq1447
seq884
seq215
seq675
seq416
seq340
seq267
seq453
seq432
seq1161
seq1237
seq543
seq1174
seq1318
seq1280
seq1505
seq1599
seq2002
seq1057
seq2050
seq1962
seq1792
seq1687
seq1319
seq1148
seq1528
seq1580
seq1389
seq1251
seq797
seq595
seq676
seq841
seq831
seq776
seq948
seq586
seq857
seq966
seq1204

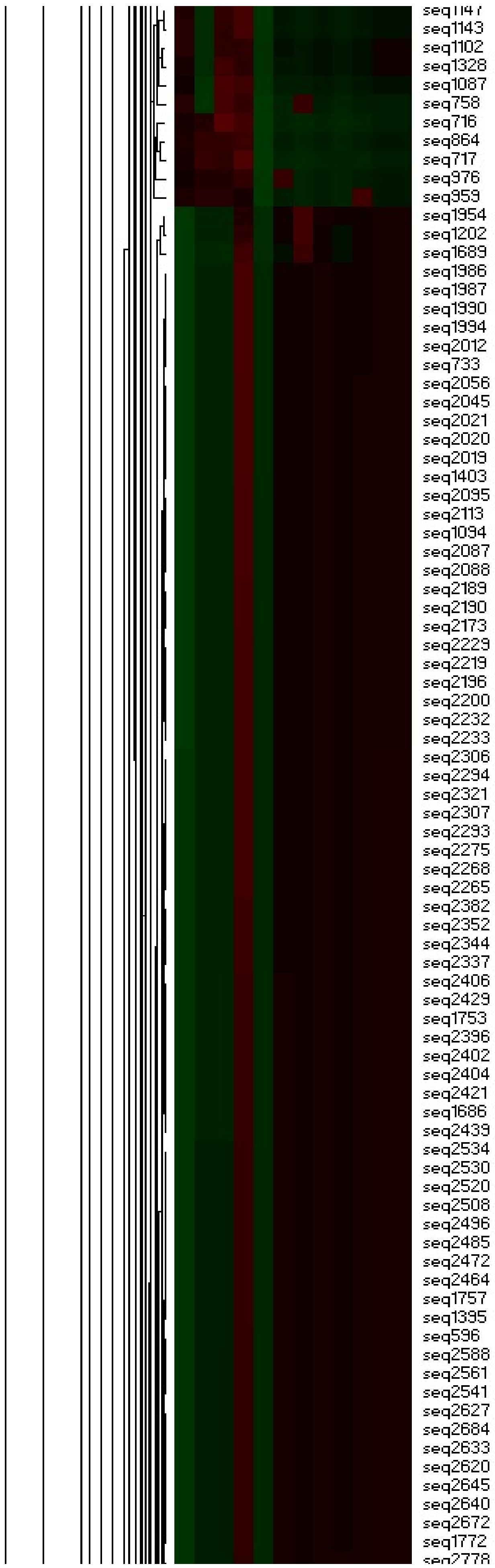
seq1147
seq1143
seq1102
seq1328
seq1087
seq758
seq716
seq864
seq717
seq976
seq959
seq1954
seq1202
seq1689
seq1986
seq1987
seq1990
seq1994
seq2012
seq733
seq2056
seq2045
seq2021
seq2020
seq2019
seq1403
seq2095
seq2113
seq1094
seq2087
seq2088
seq2189
seq2190
seq2173
seq2229
seq2219
seq2196
seq2200
seq2232
seq2233
seq2306
seq2294
seq2321
seq2307
seq2293
seq2275
seq2268
seq2265
seq2382
seq2352
seq2344
seq2337
seq2406
seq2429
seq1753
seq2396
seq2402
seq2404
seq2421
seq1686
seq2439
seq2534
seq2530
seq2520
seq2508
seq2496
seq2485
seq2472
seq2464
seq1757
seq1395
seq596
seq2588
seq2561
seq2541
seq2627
seq2684
seq2633
seq2620
seq2645
seq2640
seq2672
seq1772
seq2778

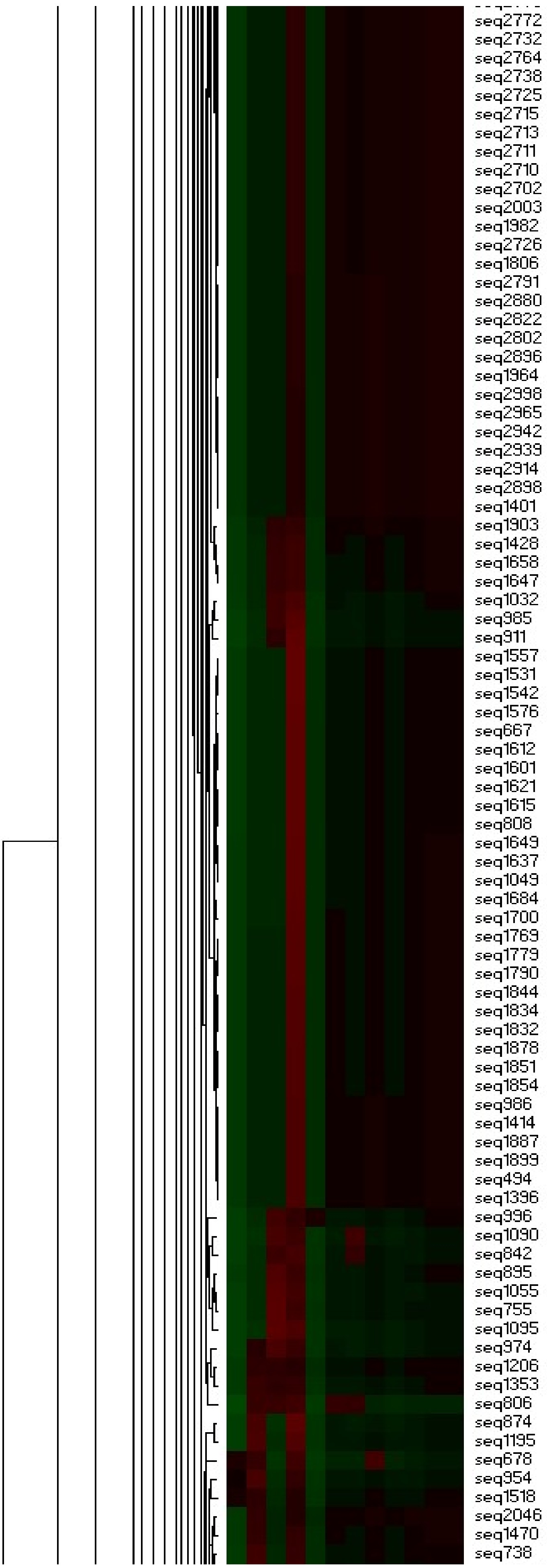
seq2772
seq2732
seq2764
seq2738
seq2725
seq2715
seq2713
seq2711
seq2710
seq2702
seq2003
seq1982
seq2726
seq1806
seq2791
seq2880
seq2822
seq2802
seq2896
seq1964
seq2998
seq2965
seq2942
seq2939
seq2914
seq2898
seq1401
seq1903
seq1428
seq1658
seq1647
seq1032
seq985
seq911
seq1557
seq1531
seq1542
seq1576
seq667
seq1612
seq1601
seq1621
seq1615
seq808
seq1649
seq1637
seq1049
seq1684
seq1700
seq1769
seq1779
seq1790
seq1844
seq1834
seq1832
seq1878
seq1851
seq1854
seq986
seq1414
seq1887
seq1899
seq494
seq1396
seq996
seq1090
seq842
seq895
seq1055
seq755
seq1095
seq974
seq1206
seq1353
seq806
seq874
seq1195
seq678
seq954
seq1518
seq2046
seq1470
seq738

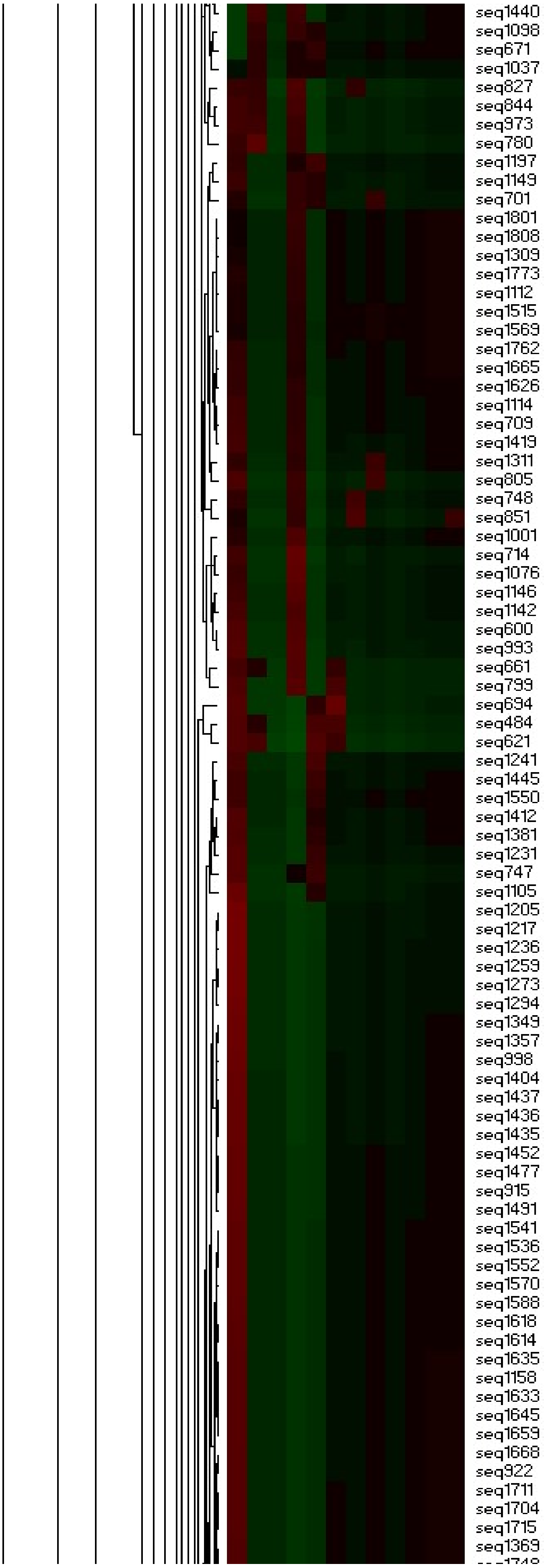
seq1440
seq1098
seq671
seq1037
seq827
seq844
seq973
seq780
seq1197
seq1149
seq701
seq1801
seq1808
seq1309
seq1773
seq1112
seq1515
seq1569
seq1762
seq1665
seq1626
seq1114
seq709
seq1419
seq1311
seq805
seq748
seq851
seq1001
seq714
seq1076
seq1146
seq1142
seq600
seq993
seq661
seq799
seq694
seq484
seq621
seq1241
seq1445
seq1550
seq1412
seq1381
seq1231
seq747
seq1105
seq1205
seq1217
seq1236
seq1259
seq1273
seq1294
seq1349
seq1357
seq998
seq1404
seq1437
seq1436
seq1435
seq1452
seq1477
seq915
seq1491
seq1541
seq1536
seq1552
seq1570
seq1588
seq1618
seq1614
seq1635
seq1158
seq1633
seq1645
seq1659
seq1668
seq922
seq1711
seq1704
seq1715
seq1369

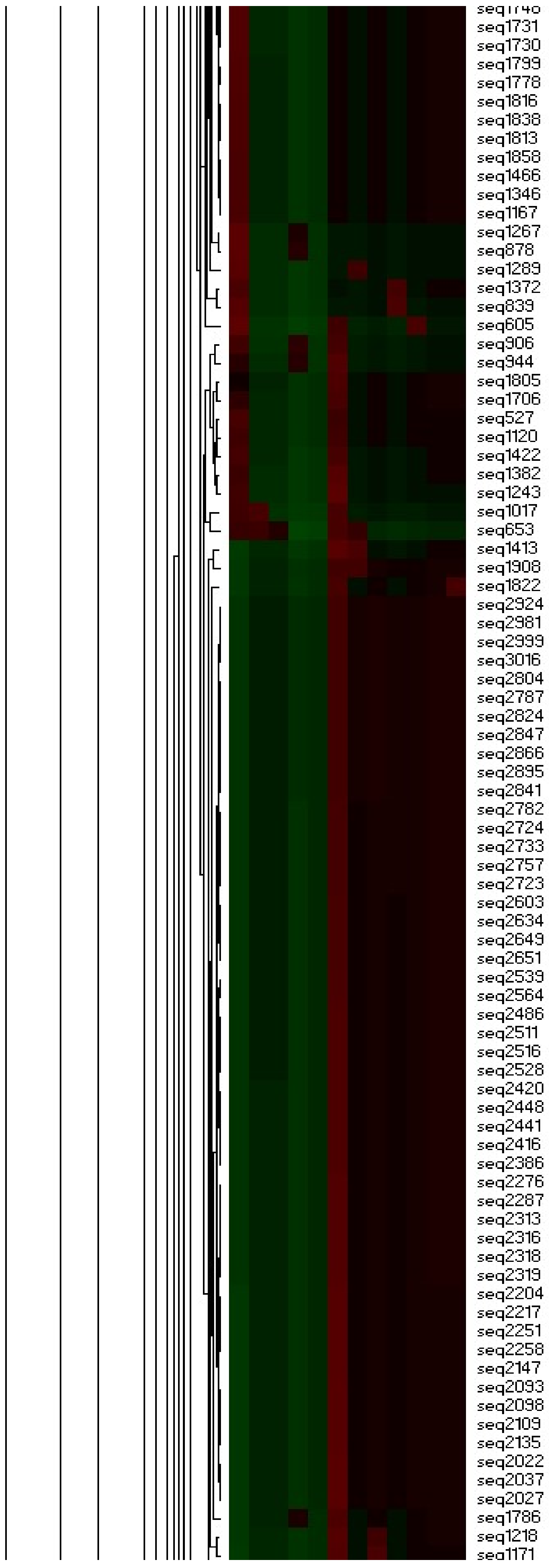
seq1731
seq1730
seq1799
seq1778
seq1816
seq1838
seq1813
seq1858
seq1466
seq1346
seq1167
seq1267
seq878
seq1289
seq1372
seq839
seq605
seq906
seq944
seq1805
seq1706
seq527
seq1120
seq1422
seq1382
seq1243
seq1017
seq653
seq1413
seq1908
seq1822
seq2924
seq2981
seq2999
seq3016
seq2804
seq2787
seq2824
seq2847
seq2866
seq2895
seq2841
seq2782
seq2724
seq2733
seq2757
seq2723
seq2603
seq2634
seq2649
seq2651
seq2539
seq2564
seq2486
seq2511
seq2516
seq2528
seq2420
seq2448
seq2441
seq2416
seq2386
seq2276
seq2287
seq2313
seq2316
seq2318
seq2319
seq2204
seq2217
seq2251
seq2258
seq2147
seq2093
seq2098
seq2109
seq2135
seq2022
seq2037
seq2027
seq1786
seq1218
seq1171

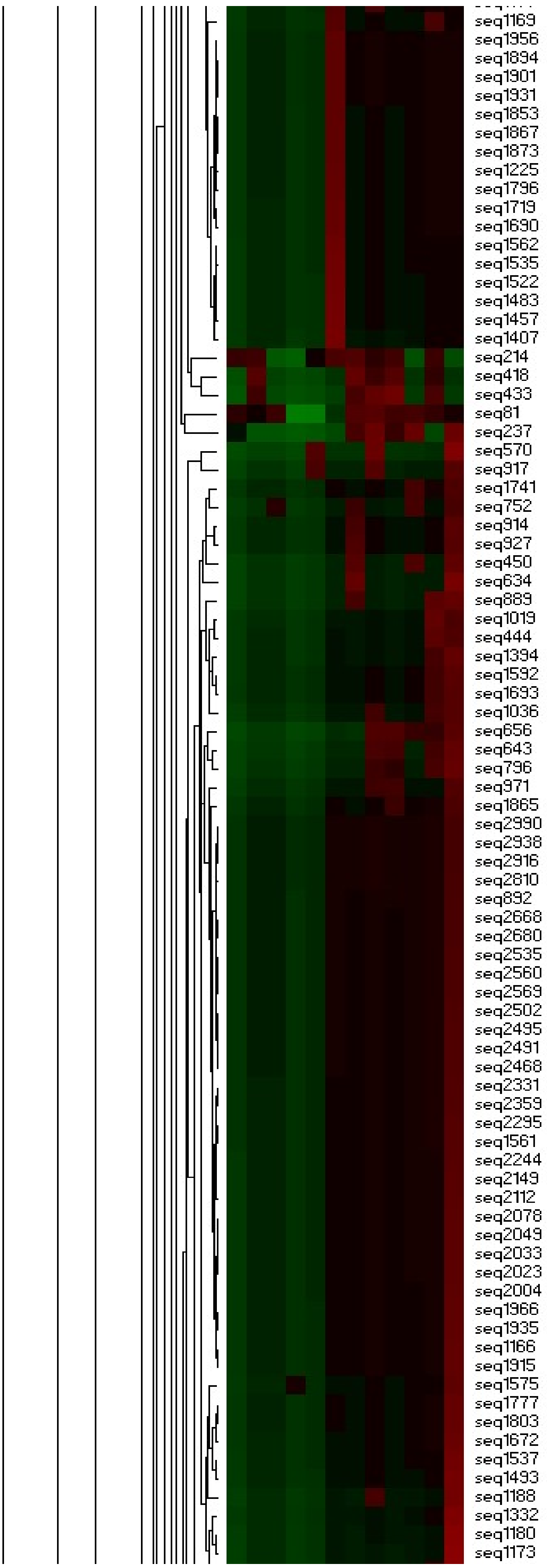
seq1169
seq1956
seq1894
seq1901
seq1931
seq1853
seq1867
seq1873
seq1225
seq1796
seq1719
seq1690
seq1562
seq1535
seq1522
seq1483
seq1457
seq1407
seq214
seq418
seq433
seq81
seq237
seq570
seq917
seq1741
seq752
seq914
seq927
seq450
seq634
seq889
seq1019
seq444
seq1394
seq1592
seq1693
seq1036
seq656
seq643
seq796
seq971
seq1865
seq2990
seq2938
seq2916
seq2810
seq892
seq2668
seq2680
seq2535
seq2560
seq2569
seq2502
seq2495
seq2491
seq2468
seq2331
seq2359
seq2295
seq1561
seq2244
seq2149
seq2112
seq2078
seq2049
seq2033
seq2023
seq2004
seq1966
seq1935
seq1166
seq1915
seq1575
seq1777
seq1803
seq1672
seq1537
seq1493
seq1188
seq1332
seq1180
seq1173

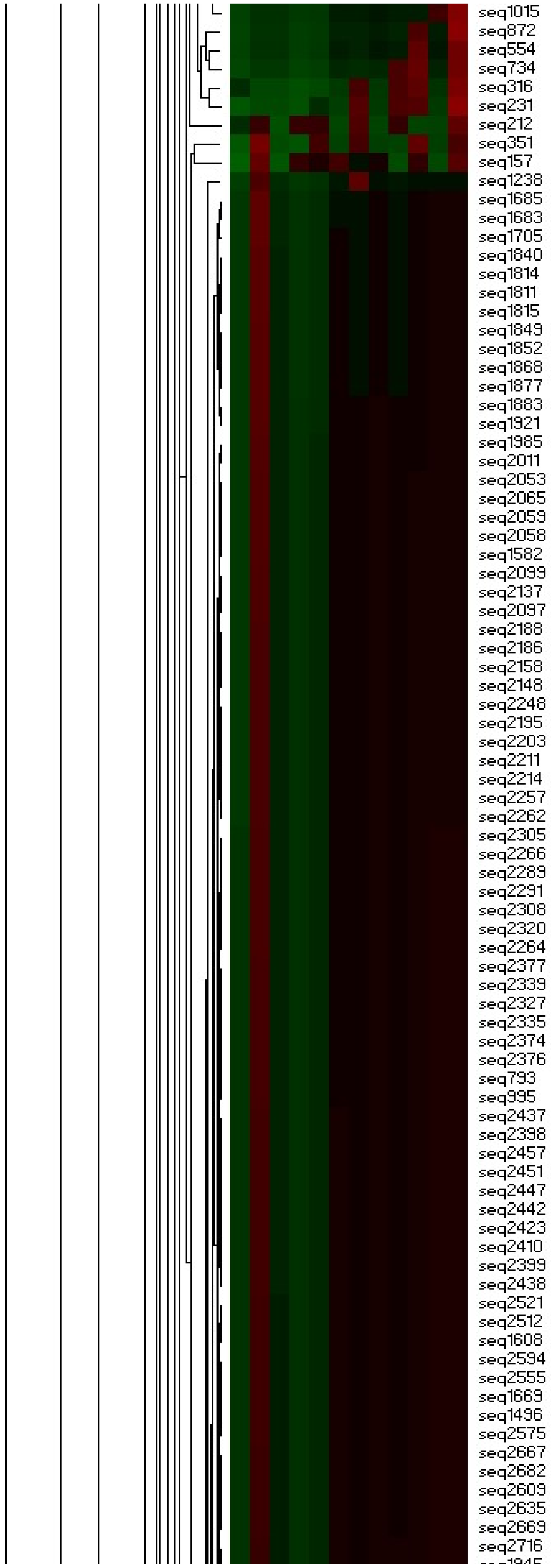
seq1015
seq872
seq554
seq734
seq316
seq231
seq212
seq351
seq157
seq1238
seq1685
seq1683
seq1705
seq1840
seq1814
seq1811
seq1815
seq1849
seq1852
seq1868
seq1877
seq1883
seq1921
seq1985
seq2011
seq2053
seq2065
seq2059
seq2058
seq1582
seq2099
seq2137
seq2097
seq2188
seq2186
seq2158
seq2148
seq2248
seq2195
seq2203
seq2211
seq2214
seq2257
seq2262
seq2305
seq2266
seq2289
seq2291
seq2308
seq2320
seq2264
seq2377
seq2339
seq2327
seq2335
seq2374
seq2376
seq793
seq995
seq2437
seq2398
seq2457
seq2451
seq2447
seq2442
seq2423
seq2410
seq2399
seq2438
seq2521
seq2512
seq1608
seq2594
seq2555
seq1669
seq1496
seq2575
seq2667
seq2682
seq2609
seq2635
seq2669
seq2716

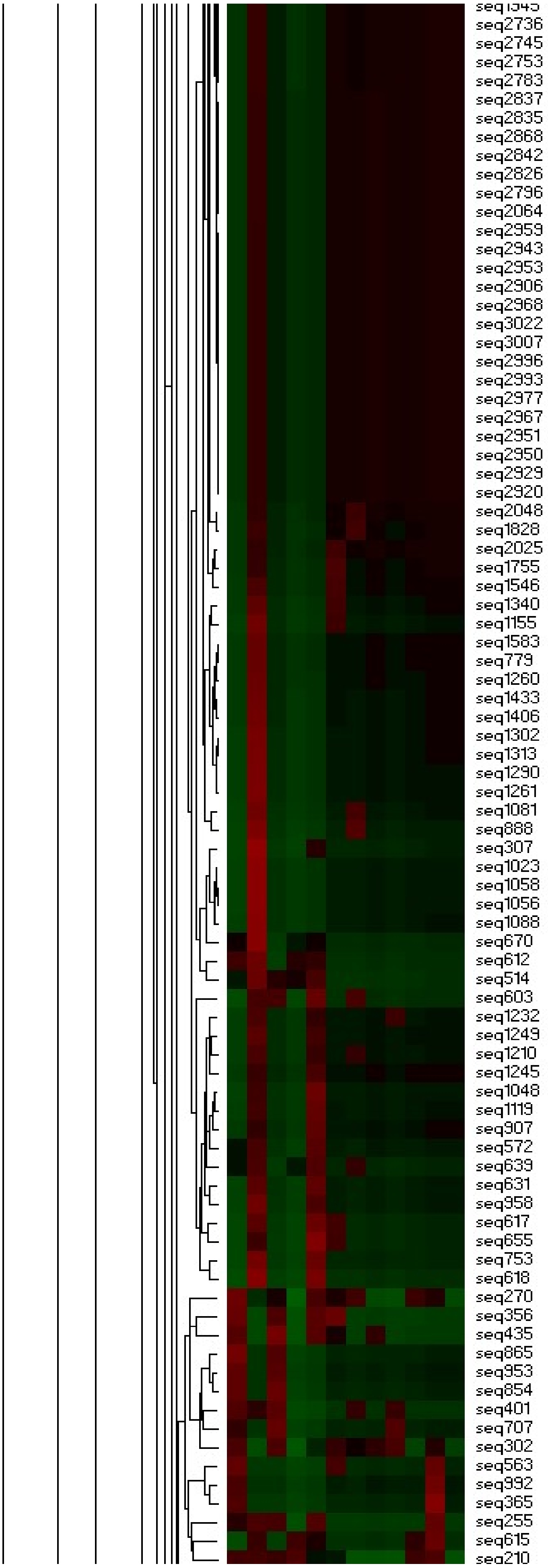
seq2736
seq2745
seq2753
seq2783
seq2837
seq2835
seq2868
seq2842
seq2826
seq2796
seq2064
seq2959
seq2943
seq2953
seq2906
seq2968
seq3022
seq3007
seq2996
seq2993
seq2977
seq2967
seq2951
seq2950
seq2929
seq2920
seq2048
seq1828
seq2025
seq1755
seq1546
seq1340
seq1155
seq1583
seq779
seq1260
seq1433
seq1406
seq1302
seq1313
seq1290
seq1261
seq1081
seq888
seq307
seq1023
seq1058
seq1056
seq1088
seq670
seq612
seq514
seq603
seq1232
seq1249
seq1210
seq1245
seq1048
seq1119
seq907
seq572
seq639
seq631
seq958
seq617
seq655
seq753
seq618
seq270
seq356
seq435
seq865
seq953
seq854
seq401
seq707
seq302
seq563
seq992
seq365
seq255
seq615
seq210

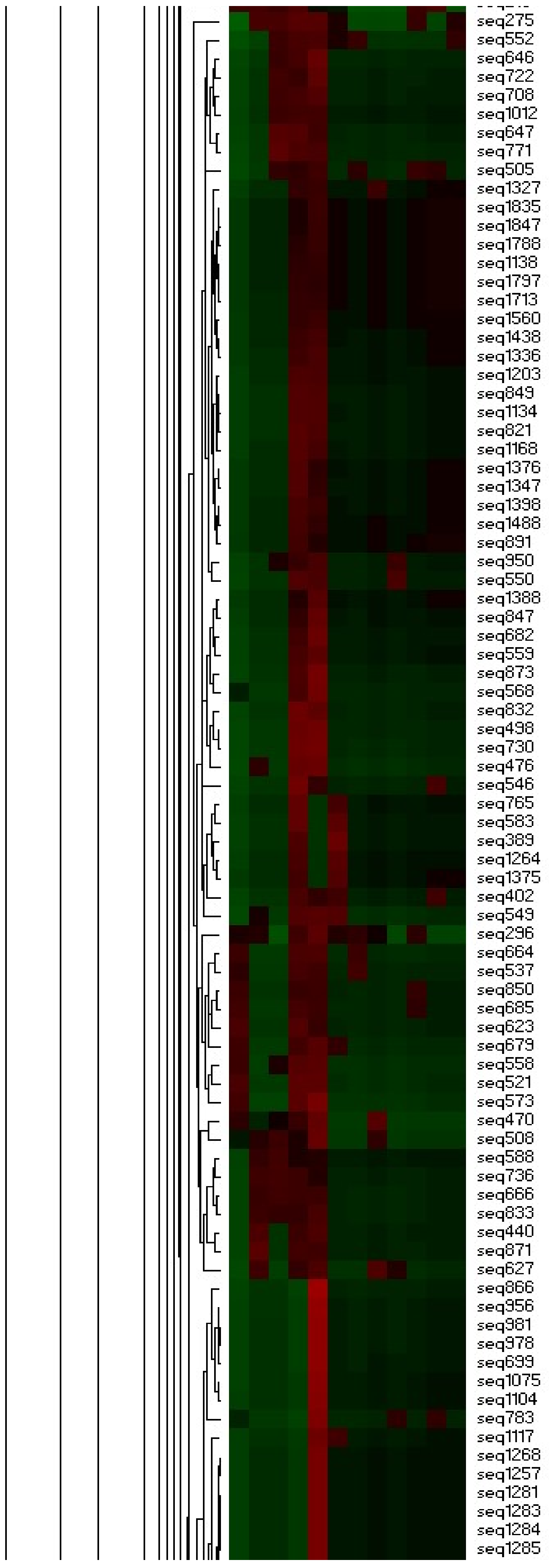
seq275
seq552
seq646
seq722
seq708
seq1012
seq647
seq771
seq505
seq1327
seq1835
seq1847
seq1788
seq1138
seq1797
seq1713
seq1560
seq1438
seq1336
seq1203
seq849
seq1134
seq821
seq1168
seq1376
seq1347
seq1398
seq1488
seq891
seq950
seq550
seq1388
seq847
seq682
seq559
seq873
seq568
seq832
seq498
seq730
seq476
seq546
seq765
seq583
seq389
seq1264
seq1375
seq402
seq549
seq296
seq664
seq537
seq850
seq685
seq623
seq679
seq558
seq521
seq573
seq470
seq508
seq588
seq736
seq666
seq833
seq440
seq871
seq627
seq866
seq956
seq981
seq978
seq699
seq1075
seq1104
seq783
seq1117
seq1268
seq1257
seq1281
seq1283
seq1284
seq1285

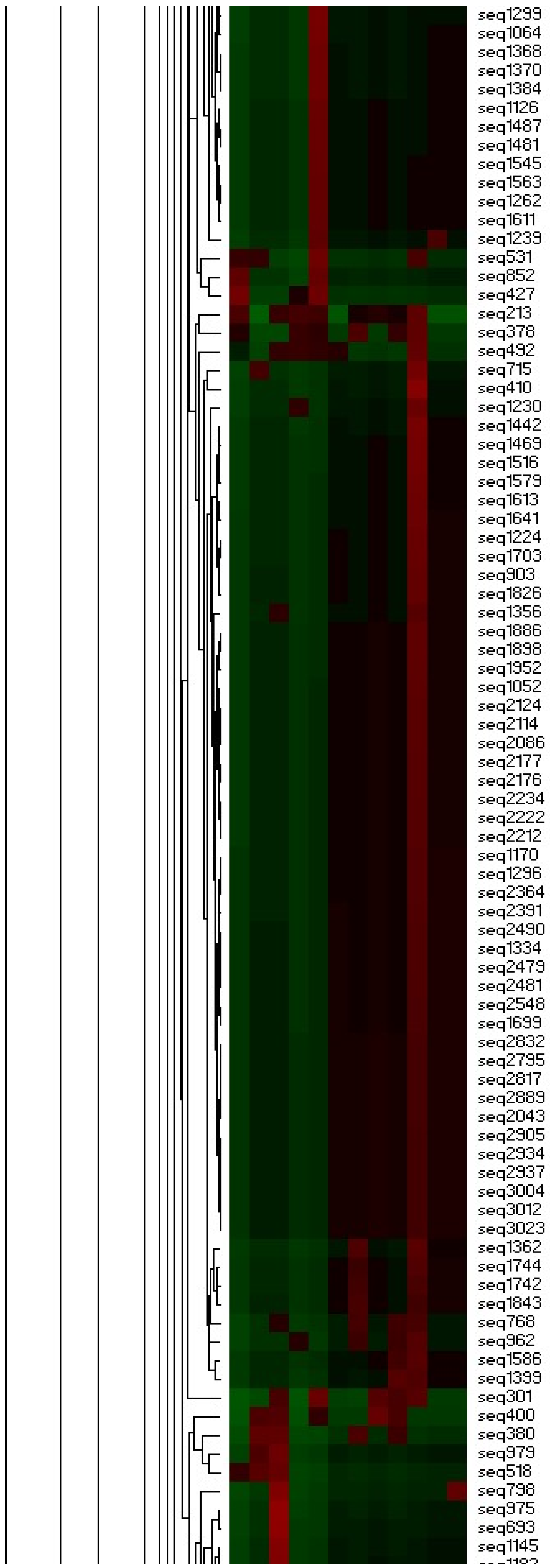
seq1299
seq1064
seq1368
seq1370
seq1384
seq1126
seq1487
seq1481
seq1545
seq1563
seq1262
seq1611
seq1239
seq531
seq852
seq427
seq213
seq378
seq492
seq715
seq410
seq1230
seq1442
seq1469
seq1516
seq1579
seq1613
seq1641
seq1224
seq1703
seq903
seq1826
seq1356
seq1886
seq1898
seq1952
seq1052
seq2124
seq2114
seq2086
seq2177
seq2176
seq2234
seq2222
seq2212
seq1170
seq1296
seq2364
seq2391
seq2490
seq1334
seq2479
seq2481
seq2548
seq1699
seq2832
seq2795
seq2817
seq2889
seq2043
seq2905
seq2934
seq2937
seq3004
seq3012
seq3023
seq1362
seq1744
seq1742
seq1843
seq768
seq962
seq1586
seq1399
seq301
seq400
seq380
seq979
seq518
seq798
seq975
seq693
seq1145

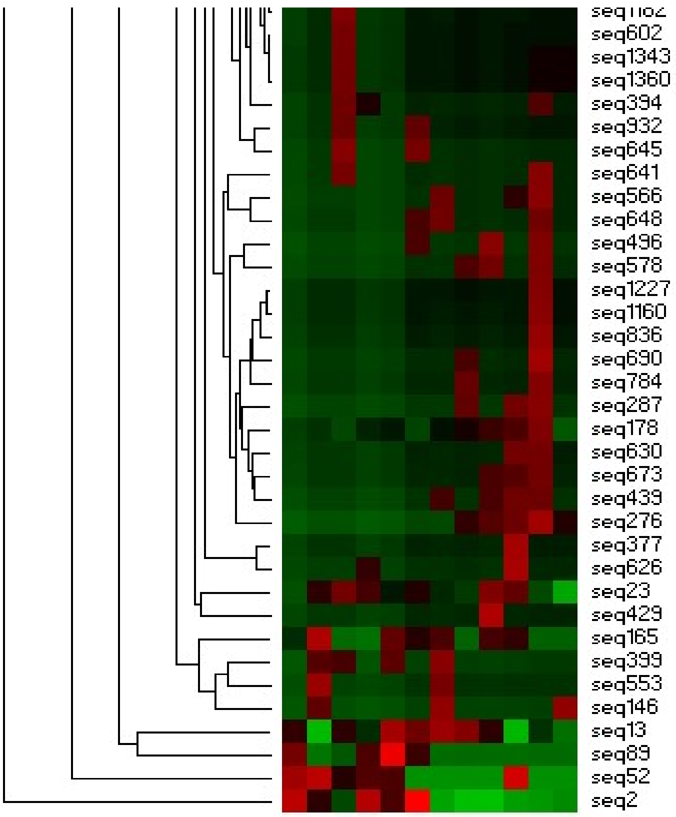
seq1162
seq602
seq1343
seq1360
seq394
seq932
seq645
seq641
seq566
seq648
seq496
seq578
seq1227
seq1160
seq836
seq690
seq784
seq287
seq178
seq630
seq673
seq439
seq276
seq377
seq626
seq23
seq429
seq165
seq399
seq553
seq146
seq13
seq89
seq52
seq2
